# Impact of host physiology and external stressors on the bacterial community of *Schmidtea mediterranea*

**DOI:** 10.1038/s41598-025-86920-0

**Published:** 2025-02-05

**Authors:** Karolien Bijnens, Sofie Thijs, Rossella Alfano, Breanne McAmmond, Jonathan Van Hamme, Tom Artois, Michelle Plusquin, Jaco Vangronsveld, Karen Smeets

**Affiliations:** 1 Centre for Environmental Sciences, Zoology, Biodiversity and Toxicology, Hasselt University, Diepenbeek, Belgium; 2 Centre for Environmental Sciences, Environmental Biology, Hasselt University, Diepenbeek, Belgium; 3 Centre for Environmental Sciences, Epidemiology, Hasselt University, Diepenbeek, Belgium; 4 Department of Biological Sciences, Thompson Rivers University, Kamloops, BC Canada; 5 Department of Plant Physiology and Biophysics, Institute of Biology and Biotechnology, Maria Skłodowska-Curie University, Lublin, Poland

**Keywords:** Host-microorganism association, Planaria, *Schmidtea mediterranea*, Variability, Environmental sciences, Microbial communities

## Abstract

To fully comprehend host-microorganism interactions, it is crucial to understand the composition and diversity of the microbiome, as well as the factors that shape these characteristics. We investigated microbiome variation using the freshwater planarian *Schmidtea mediterranea*, an invertebrate model in regeneration biology and (eco-)toxicology, by exposing the organisms to various controlled conditions. The microbiome composition exhibited high variability, with most of the bacteria belonging to the Betaproteobacteria. Among the diverse microbial communities, a few genera, such as *Curvibacter*, were consistently present, but exhibited significant alterations in response to changing conditions. The relative abundance of *Curvibacter* fluctuated during the regeneration process, initially increasing before returning to a composition similar to the beginning situation. After applying external stress, the relative abundance of *Curvibacter* and other genera decreased. Variation over time, between different origin laboratories and between individuals, showed that additional, yet to-be-identified, factors of variation are present. Taking all results together, our study provides a solid basis for future research focusing on bacterial functionality in planarians and other invertebrates.

**Supplementary Information:**

The online version contains supplementary material available at 10.1038/s41598-025-86920-0.

## Introduction

It is often stated that life cannot exist without microbes^1,2^. All animals interact with bacteria, viruses, fungi and bacteriophages, at least for part of their lives^3^. To date, our knowledge of host-microbiome interactions is limited to only a small number of animal species. Particularly for invertebrates, which comprise approximately 97% of existing animal species on Earth (IUCN), there is a significant lack of information regarding host-microorganism interactions. The wide variety of host-microorganism interactions across different animal species suggests that both the nature of the bacteria and the degree of host dependency vary significantly throughout the animal kingdom^3^. Some interactions include stable resident bacteria with distinct functions (e.g. in humans and mice), while other interactions are rather transient (e.g. in some ant species and caterpillars).

Given the large abundance of invertebrates, disturbances in their microbiomes can affect host population dynamics with far-reaching ecosystem implications due to their important roles in pollination, natural pest control, water and soil quality, and trophic cascades. Additionally, host-microorganism interactions vary with host life cycle and host tissue. Therefore, microbiome studies across a variety of invertebrates are essential to fully grasp how external factors (e.g. diet, pollutants, temperature or drought) affect microbiota and, in turn, influence host physiology and fitness^4^. To gain a comprehensive understanding of how multicellular organisms function in their environment, we need to investigate the nature and function of the associated microbiomes, as well as the variation within these microbiomes.

Here, we studied the associated bacteria of the planarian *Schmidtea mediterranea*, a freshwater flatworm with remarkable regenerative properties. After loss or damage, this organism can restore all its tissues, including its central nervous system in approximately 7–10 days^5–8^. The processes of tissue development and regeneration can be artificially induced by amputation and, as such, planarians are commonly used as models in developmental and regeneration research^9^. In (eco)toxicology planarians are used to monitor water quality or study the toxicity mechanisms of aquatic pollutants^7,10–17^. Given that it is easy to artificially induce changes in their environment, the effects on microbiome variation can be easily studied in relation to their physiology. In this regard, studies published by Arnold et al.^18^ and Lee et al.^19^ showed that disturbances in planarian microbiome composition are linked with regeneration defects. In addition, we found that exposure to silver nanoparticles caused regenerative defects^11^ and compositional shifts in the planarian microbiome^20^.

To fully understand the role of the microbiome in animal physiology, it is essential to characterize healthy microbiomes in different organisms, physiological states and during external fluctuations. We addressed the variability of the planarian microbiome by experimentally inducing different condition to investigate microbiome variation in physiological state, after an alternative diet and after exposure to external stressors. In addition, we evaluated microbiome composition over time, and compared the microbiomes between individuals, different laboratories of origin and different planarian species. We report the microbiome variation as it is present in a regular laboratory environment, including known and unknown drivers of variation. This research is among the first to compare that many conditions, which is a step forward to better understand host-microbiome functionality. 

## Materials and methods

### Planarian husbandry

The majority of the experiments were performed using an asexual laboratory strain of the planarian *S. mediterranea*, cultivated in planarian medium consisting of 1.6 mM NaCl, 1 mM CaCl_2_, 1 mM MgSO_4_, 0.1 mM MgCl_2_, 0.1 mM KCl and 1.2 mM NaHCO_3_ in milliQ water^21^. Worms were kept in the dark at a constant temperature of 20 °C and fed once a week with small pieces of frozen veal liver, which were kept at -25 °C. After four hours of feeding, the liver was removed, the planarians were rinsed twice with distilled water and fresh planarian medium was added. Worms of similar sizes (approximately 4–6 mm) were selected for experiments and starved for at least seven days before the experiment, unless stated otherwise. Within the planarian community, it is generally accepted that at least 7 days of starvation prior to experiments is necessary to minimize interference with measurements, as this is the time needed to fully digest the present food. During experiments, the planarian medium and exposure solutions were refreshed every 2–3 days.

### Experimental design

Various research questions regarding variation in the microbiome of planaria were addressed (Table 1). Depending on the availability of the technique and the research question, the factors of variation were investigated using 16 S rRNA gene amplicon sequencing (referred to as *Data_experiment* in Table 1 and throughout the text) or bacterial cultivation with subsequent identification via Sanger sequencing of the partial 16 S rRNA gene. The bacterial cultivation is included to compare to previous studies that studied the cultivable fraction of the planarian microbiome. More specifically, to identify both cultivable and non-cultivable bacteria, 16 S rRNA gene amplicon sequencing was used, while Sanger sequencing was used to identify individual colonies. Scanning electron microscopy (SEM) and fluorescent in situ hybridisation (FISH) were used to obtain spatial information on the localisation of bacteria in planarians (Section “Microscopic detection of bacteria”).

**Table 1:** Overview of the experiments conducted in this study, with associated goals and techniques.

Goal	Techniques (Dataset)	Conditions	Details
Study microbial variation during different stages of regeneration	16 S rRNA seq (*Data_Develop*)	0 dpa 3 dpa 7 dpa 14 dpa	Regenerating worms 7d starved Head + tail
Explore the effect of starvation and a different food source	16 S rRNA seq (Data_Food)	Starved Bovine liver fed Boiled egg-white fed	Adult/intact worms 14d starved 0d, 1w, 3w, 4w and 8w fed
Investigate microbial changes due to antibiotics exposure	16 S rRNA seq (*Data_Antibiotics*)	Not-exposed Gentamycin exposed Vancomycin exposed	Adult/intact worms 14d starved 14d exposed
Study microbial changes due to chemical stress	16 S rRNA seq (*Data_Chemstress*)	Not-exposed Cd-exposed MMS-exposed	Adult/intact worms 7d starved 7d exposed
Study microbial changes after silver nanoparticle exposure, combined with *Data_antibiotics* and *Data_chemstress* to find recurring affected bacteria after stress	16 S rRNA seq^20^	Not-exposed AgNP-exposed	Adult/intact worms 7d starved 3d, 7d, 14d exposed
Investigate temporal variation	16 S rRNA seq (Data_Time)	2017 2018 (set 1) 2018 (set 2) 2019	Adult/intact worms 7d starved Not-exposed
Study variation between different laboratories	16 S rRNA seq (*Data_Labs*)	Lab 1 Lab 2 Lab 3	Adult/intact worms 7d starved
Compare microbiomes between different planarian species	16 S rRNA seq (*Data_Planaria*)	*Schmidtea mediterranea* *Girardia tigrina* *Dugesia* sp.	Adult/intact worms 7d starved
Locate associated bacteria in planarians	Agar cultivation/Sanger sequencing SEM/FISH	Mucus present Mucus absent	Adult/intact worms 7d starved

The purpose of these experiments was to identify all types of variability across the bacterial community in planarians. A better understanding of the stable or unstable associations allows to functionally link planarian microorganism interactions. For this reason, we used a set-up that is similar to the set-ups used in other planarian experiments, starting from non-sterilized animals, hence, allowing to assess variability. Within an experiment, all animals came from the same batch, to properly study the effect of one factor/variable. When available, we included background information regarding planarian culture medium and food source, as clarified in the supplemental methodology section (§ Bioinformatics processing). In the future, additional environmental controls can be included for all experiments to better understand the source of microbial variation. In addition, we also compared the microbiome of animals over different experiments, to raise awareness that known and unknown factors can influence the microbiome and the elicited physiological responses (e.g. variation over time, variation over different laboratories). The general idea behind this work was not to minimize variation, but to embrace it and include known and unknown factors of variation that are present in regular planarian experiments and to report on the effect on the planarian microbiome.

#### Amputation and regeneration

To assess microbiome composition and diversity during regeneration, adult worms were starved for seven days, followed by a transversal amputation anterior of the pharynx using a sterilised blade, resulting in a head fragment and a tail fragment that included the pharynx. Immediately after amputation (zero days post amputation (0 dpa)), samples were taken for Illumina sequencing. The remaining fragments were left to develop or regenerate. After three, seven or 14 days of regeneration, samples were taken for Illumina sequencing (*Data_Develop*).

#### Feeding and starvation

Two independent experiments were performed to study the effect of an alternative diet and starvation on planarian microbiome, health and physiology. In the first experiment, adult worms were starved for two weeks and then fed once a week with pieces of veal liver or boiled egg whites. Samples for Ion Torrent sequencing were taken at the beginning of the experiment (meaning after two weeks of starvation), and after one week, three weeks, four weeks and eight weeks of feeding (*Data_Food*). In a second independent experiment to relate an alternative diet to physiology, worms were starved or fed with liver or egg white during eight weeks. Each week, general behaviour and physiological changes of the worms were recorded. In addition, three pictures per individual were taken weekly using a Nikon Ds-Ri2 digital camera mounted on a Nikon SMZ800 stereomicroscope (Nikon Instruments Inc.). These pictures were used to calculate the average total body surface area of each worm using ImageJ^22^.

#### Exposures

To assess the effect of antibiotic exposure on the planarian microbiome, adult worms were starved for 14 days and then exposed for 14 days to 50 µg ml^−1^ gentamicin (gentamicin sulphate, Sigma-Aldrich Cat. No. G1914) or 25 µg ml^−1^ vancomycin (vancomycin hydrochloride, VWR Cat. No. A1839) dissolved in planarian culture medium, before sampling for Ion Torrent sequencing (*Data_Antibiotics*). Exposure concentrations were based on the study of Arnold et al. 2016^18^. To study the effect of an environmental stressor and (DNA alkylating) carcinogen, adult planaria were starved for seven days and then exposed for seven days to either 10 µM cadmium (CdCl_2_, Sigma-Aldrich Cat. No. 208299) or 50 µM methyl methanesulfonate (MMS, Sigma-Aldrich Cat. No. 129925) dissolved in planarian medium. The samples were processed for Ion Torrent sequencing (*Data_Chemstress*). The concentration of Cd used was based on previous LC_50_ determinations^23^, while the concentration of MMS used was based on previous lethality screens and found to be sublethal^14^. In particular, we chose these two components, as previous studies of our group already resulted in information about their effects on planarian physiology, allowing us to make preliminary links between the planarian microbiome and physiology under different types of stress.

#### Temporal variation

To study temporal variation (differences among multiple individuals over time), we compared 16 S rRNA gene amplicon sequences of adult, non-exposed worms sampled in 2017, 2018 (2 sets) and 2019 (*Data_Time*). All worms were kept in regular culture conditions as described in ‘Planarian husbandry’. The worms from the 2017 experiment were starved for seven days and then pooled together, resulting in five worms per sample. Worms from 2018 to 2019 were starved for seven days and individually sampled. Samples from 2017 to 2018 were prepared for Ion Torrent sequencing, while samples from 2019 were prepared for Illumina sequencing (a choice based on the availability of the platform).

#### Different laboratories

Apart from the worms cultured in our own lab (Lab 1), we received asexual individuals from the same species from other laboratories (Lab 2 located in Spain and Lab 3 located in Germany) to assess microbiome variation due to different origins. The samples were subjected to Illumina sequencing, resulting in the dataset *Data_Labs*.

#### Different species of planarians

To compare the planarian microbiome over different species, wild planarians identified as *Girardia tigrina* and individuals of *Dugesia* sp. were collected in a pond close to the campus of Diepenbeek (Belgium) and cultured in our lab, using the same culturing conditions as described in 2.1 (planarian husbandry). The samples were subjected to Illumina sequencing, resulting in the dataset *Data_Planaria*.

### High-throughput sequencing

For the amplicon sequencing experiments, we studied worms at an individual level, as whole organisms or fragments, including their internal and external associated bacteria. Before sampling in liquid nitrogen, individuals were rinsed three times in sterile planarian medium to remove loosely associated bacteria and study the tightly associated external and internal bacteria. Samples were stored at -74 °C and processed further within two months to ensure good quality. We used an optimised phenol-chloroform DNA extraction protocol described by Bijnens et al. 2021^20^ to obtain DNA, as also described in Supplementary material 2 (§ DNA extraction). Using the Illumina and Ion Torrent sequencing platform, the microbiome was characterised by targeted gene amplification with primers targeting the 16 S rRNA V3-V4 region, and platform specific adaptors and barcodes following the protocol as further described in Supplementary material 2 (§ Library preparation). The Ion Torrent library preparation was based on the primers 341 F and 806R (read size: 1 × 400 bp). For the Illumina sequencing, primers 341 F and 785R^24^ were used (read size: 2 × 300 bp). After demultiplexing the reads using the build-in platform software, the resulting FASTQ files were denoised using Dada2 in RStudio 1.2.1335, R version 3.5.3 as described in Supplementary material 2 (§ Bioinformatics processing). The number of highly qualitative reads are summarized in Table S1 (Supplementary material 3). The resulting ASV table with taxonomy and mapping file can be found in Supplementary material 1. A detailed description of the following downstream taxonomic analyses can be found in Supplementary material 2 (§ Taxonomic analyses). Based on the resulting 16 S rRNA sequences, a functional prediction was performed in MicrobiomeAnalyst^25^, using the KEGG database^26–28^, as described in Supplementary material 2 (§ Functional prediction).

### Bacterial cultivation and Sanger sequencing

Individual worms were rinsed in filter-sterilised planarian medium and crushed by pipetting up and down in 250 µl planarian medium. Then 100 µl was spread out an agar plate containing 869 rich medium (5 g yeast extract, 10 g tryptone, 1 g D-glucose, 5 g NaCl, 0.3 g CaCl_2_.2H_2_O and 15 g agar per liter, pH 7), that was incubated at 30°C for 48 hours. Individual colonies were picked, purified and stored at − 45 °C, in a 15% w/v glycerol solution with 0.85% w/v NaCl. DNA was extracted from the isolates, PCR-amplified with 26F (5’-AGAGTTTGATCCTGGCTCAG-3’) and 1391R (5’-ACGGGCGGTGTGTRC-3’) primers as described previously^29^ and subjected to Sanger sequencing (Macrogen, Netherlands). The sequences were inspected and quality trimmed using Chromas (Technelysium, DNA Sequencing Software) and classified using EZBioCloud^30^. Fresh planarian medium (i.e. planarian medium that did not come into contact with worms) did not result in colonies.

### Microscopic detection of bacteria

To obtain spatial information on the bacteria associated with *S. mediterranea*, the bacteria were visualised in whole animals.

For scanning electron microscopy, worms were rinsed in ice-cold filter-sterilised planarian medium and then individually fixed overnight at 4 °C in Karnovsky fixative, containing 0.1 M cacodylate buffer (pH 7.4), 2% paraformaldehyde and 2.5% glutaraldehyde. Next, worms were washed with a sodium-phosphate buffer (0.2 M Na_2_HPO_4_.2H_2_O and 0.2 M NaH_2_PO_4_.2H_2_O, pH 7) for 1 h, followed by a 3 times 10 min rinse in distilled water. The samples were then dehydrated in 50%, 70%, 80%, 90% and 95% ethanol solutions, each for 15 min, followed by 3 times 15 min in 100% ethanol. After 10 min of air-drying the samples, a drop of tert-butyl alcohol was added, followed by overnight air-drying. Samples were mounted, gold-coated by the automated sputter coater JEOL JFC-1300 at 30 mA for 15 s, and then scanned using a Hitachi TM3000.

For fluorescent in situ hybridisation, the mucus of the worms was removed using an 8 min incubation in 5% NAC dissolved in phosphate buffered saline (PBS), followed by a brief wash in PBS with 0.3% Triton X-100 (PBST). Then, the worms were fixed at room temperature (RT) for 20 min in 4% formaldehyde (in PBS). Formaldehyde fixation strengthens the cell wall of Gram-negative bacteria, but renders Gram-positive bacteria impermeable. To stain the latter, an ethanol-based fixation is needed, but, as the microbiome of planaria is mainly composed of Gram-negative bacteria, we focused on these. After fixation, the worms were incubated for three 10 min incubations in PBST and then dehydrated in steps towards 100% methanol. A 1-hour incubation in methanol at -20 °C was followed by bleaching the worms overnight in 6% hydrogen peroxide (in methanol) by illumination with a cold lamp of approximately 3000 K. The next day, the samples were rinsed with methanol for three 10 min intervals at room temperature, followed by re-hydratation towards PBST. Each re-hydratation step took 10 minutes and was performed at room temperature. The planarian tissue was then incubated in pre-heated (37 °C) reduction solution (50 mM DTT, 1% NP-40 and 0.5% SDS) for 6 min until an intestinal pattern appeared. After three 10 min incubations in PBST, the worms were permeabilised for 6 min in pre-heated (37 °C) proteinase K solution (20 µg/ml in PBST), followed by three quick washes in PBST and a 10 min post-fixation in 4% formaldehyde at RT. After three 10 min incubations in PBST, the bacterial cell wall was permeabilised for 3 min each in 50%, 70% and 96% ethanol. The ethanol was removed and compressed air was used to dry the samples. To target most bacteria, the EUB388 probe (5’-GCTGCCTCCCGTAGGAGT-3’) was added to the hybridization buffer (900 mM NaCl, 20 mM Tris/HCl (pH 8), 30% deionised formamide and 0.01% SDS), which was added to the planarian tissue prior to incubation for 3 h at 46 °C in the dark. The samples were then rinsed twice with washing buffer, followed by a 15 min incubation in washing buffer at 47 °C in the dark. Finally, the samples were rinsed twice in ice-cold milliQ water, dried with compressed air and then mounted in glycerol. After clearing at 4 °C, the samples were imaged using a Nikon Ds-Ri2 camera mounted on a Nikon eclipse i80 fluorescence microscope.

### Sequencing datasets

The 16 S rRNA gene amplicon sequencing data were submitted to the Short Read Archive of NCBI, accessible via PRJNA1036534. To find recurring microbial changes under environmental stress, we also included data of our previous study^20^, available via PRJNA675880, which describes the effects of silver nanoparticles (AgNP) on the planarian microbiome.

### Figure generation

Graphs were generated in R in SVG format, Excel 2016, Prism 5 or Past and then further modified (i.e. font enlargement, consistent colouring) in Adobe Illustrator 2020 or Adobe Photoshop 2020. Final figures were assembled in Adobe Illustrator 2020.

## Results

We investigated the microbial composition and diversity of the freshwater planarian *S. mediterranea*. The planarian microbiome was compared under a large set of different physiological and stress conditions (Table 1). The primary objective of these experiments was to elucidate the variability within the bacterial community of planarians. We examined both known and unknown factors that contribute to variation in standard planarian experiments, and we assessed their impact on the planarian microbiome. In doing so, we aimed (1) to identify both stably associated bacterial taxa as well as unstable associations within the planarian microbiome, and (2) to correlate these variations to physiological changes to hypothesize potential bacterial functionalities.

### The microbiome is variable during the different regeneration stages

**Fig. 1:**
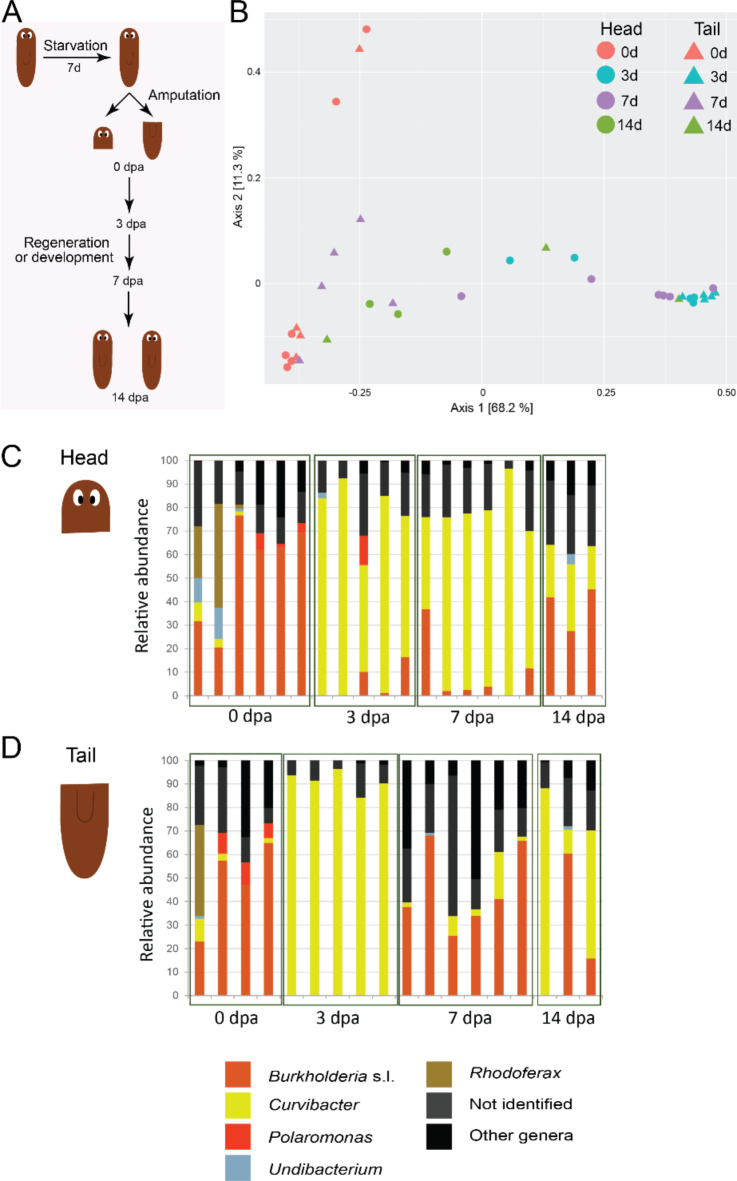
Microbial variation during regeneration. (**A**) Experimental setup for the *Data_Develop* sequencing data. Adult worms were starved for 7 days and then amputated in head and tail fragments. Right after amputation (0 dpa) samples for sequencing were taken and compared with samples taken after 3, 7 and 14 days of regeneration or development. (**B**) PCoA of weighted UniFrac illustrating beta-diversity between the different regeneration stages in head and tail fragments (*p-adj* = 0.001, *R*^2^ = 0.459). (**C**) Relative abundances at the genus level per individual during the different stages of regeneration, specific to regenerating head fragments. (**D**) Relative abundances at genus level per individual during the different stages of regeneration, specific to regenerating tail fragments. (PCoA: principal coordinate analyses, RA: relative abundance, dpa; days post amputation, head 0 dpa *n* = 6, head 3 dpa *n* = 5, head 7 dpa *n* = 6, head 14 dpa *n* = 3, tail 0 dpa *n* = 4, tail 3 dpa *n* = 5, tail 7 dpa *n* = 6, tail 14 dpa *n* = 3).

Planarians are characterized by a high regenerative capacity and are known to functionally restore missing body parts and tissues in a very short timeframe. The regeneration process is triggered by an amputation wound, which forms a potential infection site for external bacteria. During the regeneration process, a complex network of signalling molecules, downstream pathways and molecular responses are involved, all with the potential to affect the microbiome and/or vice versa. It is already known that a disturbed microbiome affects planarian physiology, characterized by tissue lesions and regenerative defects^18^. Here, we characterised the planarian microbiome during different stages of the regeneration process to estimate if the associated bacteria are influenced during this physiological process and/or vice versa. Adult animals were amputated anterior of the pharynx, resulting in a head and tail fragment, that each regenerated their missing body parts to form a complete animal (Fig. 1A). Samples were taken for sequencing immediately after amputation (0 dpa, days post amputation) and after 3 days (3 dpa), 7 days (7 dpa) and 14 days (14 dpa) of regeneration, which represent early, late and complete regeneration stages, respectively.

During the different regeneration stages, the diversity and composition of the planarian microbiome changed, as indicated by two independent sequencing experiments. The PCoA plot based on the weighted UniFrac showed separation based on the regeneration stage (*p-adj* = 9.99e^− 4^, *R*^2^ = 0.46), partly explained by the interaction with the head or tail fragment (fragment × regeneration stage: *p- adj* = 1.99e^− 3^, *R*^2^ = 0.18), along with a primary and secondary axis explaining respectively 68.2% and 11.3% of the total variation (Fig. 1B). A third axis was plotted in Fig. S1D, E. At phylum level, mainly Proteobacteria were present in both head and tail fragments (Fig. S1A, B). In tail fragments, the Bacteroidetes content was higher at 7 dpa, compared to the other time-points and compared to head fragments. The largest differences were observed at the genus level (Fig. 1C, D). The ratio between the genera *Burkholderia* s.l. and *Curvibacter* changed during the different stages of regeneration. Immediately after amputation (0 dpa) the *Curvibacter* versus *Burkholderia* s.l. ratio was in favour of *Burkholderia* s.l., while at 3 dpa the microbiome was uniform and dominated by *Curvibacter*. In 7 dpa microbiomes, the *Curvibacter* versus *Burkholderia* s.l. ratio was dependent on the fragment, as heads were dominated by *Curvibacter* and tails by *Burkholderia* s.l. After 14 days of regeneration (14 dpa), head and tail microbiomes were more alike, although variation between the individuals was observed. Rarefaction curves are shown in Fig. S1C. A background sample was included in the analyses, describing the composition of fresh planarian medium at that specific time (Fig. S1F), with the main genera being *Variovorax* and *Pseudomonas*.

In summary, the data presented here show that regenerating worms are dominated by Proteobacteria and Bacteroidetes, similar to adult worms (Table 2)^18,20^. However, the bacterial composition fluctuates during different stages of development, especially at the genus level and more specifically in the *Curvibacter* versus *Burkholderia* s.l. ratio. Given the antifungal properties of *Curvibacter* in regenerative *Hydra*^31^, we hypothesize a similar function for *Curvibacter* in planarians, as we discuss in detail in the “Discussion” Section. Alternatively, it is possible that factors produced during the regeneration process favour a different bacterial composition.

**Table 2:** Overview of the key finding in this study.

References	Factor under study	Key findings
Section “The microbiome is variable during the different regeneration stages” Figure 1 Figure S1	Developmental stage	The composition of the associated microbiome changes during the regenerative phases. During the initial phases of the regeneration process the relative abundance of *Curvibacter* increases, while Burkholderia s.l. decreases. The composition changes again in favor of the latter in the later phases of regeneration.
Section “Changing the food source alters planarian physiology, but not profoundly the microbial composition” Figure 2 Figure S2	Alternative diet	An alternative diet does not significantly alter the microbiome composition of planarians. Other environmentally related factors play a more defining role, as the microbiome after 8 weeks differs from the other timepoints.
Section “External stressors influence the microbial composition” Figure 3 Figure S3	Exposure to antibiotics	Exposure to antibiotics targeting Gram-negative and Gram-positive bacteria influences the planarian microbiome. The relative abundance of several genera is affected, some specific for the antibiotic uses, while others are affected in both conditions.
Section “External stressors influence the microbial composition” Figure 3 Figure S3	Exposure to chemical stressors	Exposure to chemical stressors influences the planarian microbiome; i.e. the relative abundance of Firmicutes and Gammaproteobacteria decreased, with a concomitant increase in Bacteroidetes.
Section “External stressors influence the microbial composition” Figure 4	Recurring patterns after environmental stress	After environmental stress several genera are recurrently affected, including *Curvibacter*, *Ca. Symbiobacter*, *Undibacterium* and *Acidovorax*.
Section “The microbiome of S. mediterranea is variable over time and over different laboratories” Figure 5	Temporal variation	Temporal variation is present, both in composition as in diversity. The most abundant phylum was Proteobacteria in all conditions, although the proportion of Bacteroidetes varied.
Section “The microbiome of S. mediterranea is variable over time and over different laboratories” Figure 6 Figure S5	Origin laboratories	At higher taxonomic level, the ratio of Proteobacteria vs. Bacteroidetes differs depending on the origin laboratory. At lower taxonomic level (i.e. genus) differences were more pronounced.
Section “The microbiomes of closely related planarian species share bacterial taxa” Figure 7 Figure S6	Planarian species	Closely related planarian species share bacterial taxa and are also dominated by Proteobacteria and Bacteroidetes, although their specific ratios vary.
Section “The microbiome of S. mediterranea is highly variable, although a set of three genera is consistently present in adult animals”	Commonly associated bacteria	Relative abundances at higher taxonomic levels (e.g. Proteobacteria and Bacteroidetes) remain similar over multiple conditions. Within healthy individuals, three common genera were detected: *Curvibacter*, *Staphylococcus* and *Sphingomonas*.
Section “The planarian microbiome might be important for the metabolism of biomolecules” Figures S7–S8	Functional prediction of associated bacteria	Based on a predictive analyses (with known limitations and no definitive conclusions) we found altered predicted genes involved in the metabolism of biomolecules after exposure to stressors.
Section “The planarian microbiome is present in the gut” Figure S9	Location of bacteria	Bacteria were detected in the planarian gut.
Multiple	Individual variation	Variation between individuals is present.

### Changing the food source alters planarian physiology, but not profoundly the microbial composition

**Fig. 2:**
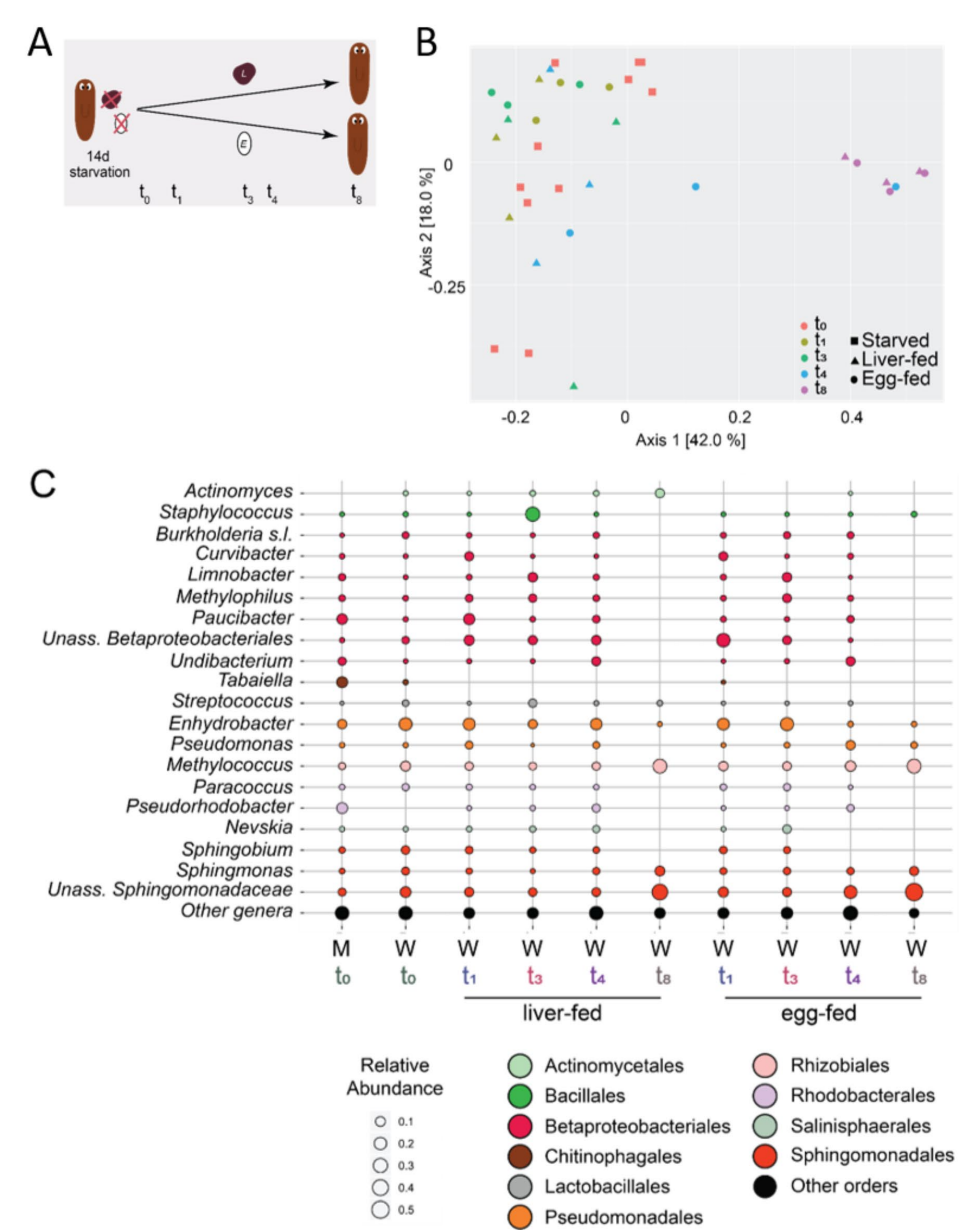
Microbial variation due to an alternative diet. (**A**) Experimental setup for the *Data_Food* sequencing data. Adult worms were starved for 14 days and then sampled for sequencing (t_0_). The remaining worms were fed with liver or egg-whites during 1 week (t_1_), 3 weeks (t_3_), 4 weeks (t_4_) and 8 weeks (t_8_). (**B**) PCoA of weighted UniFrac illustrating beta-diversity between the different conditions, including starvation, liver-fed and egg-fed worms (Food source: *ns*, *R*^2^ = 0.02, Time feeding: *p-adj* = 9.99e^− 4^, *R*^2^ = 0.42, heterogeneity of variances). (**C**) Average relative abundances at the genus level at the different time points for the different conditions. The genera are coloured by their corresponding orders and the size of the bubble corresponds to the relative abundance. (W: worms, M: exposure medium that came into contact with the worm, PCoA: principal coordinate analyses, RA: relative abundance, t_0_ M *n* = 4, t_0_ W *n* = 4, t_1_ liver-fed *n* = 3, t_1_ egg-fed *n* = 3, t_3_ liver-fed *n* = 3, t_3_ egg-fed *n* = 3, t_4_ liver-fed *n* = 3, t_4_ egg-fed *n* = 3, t_8_ liver-fed *n* = 3, t_8_ egg-fed *n* = 3)

In other studies, diet has been found to be a determining factor in microbiome composition and diversity^32^. Here, in order to investigate the effect of an alternative food source and starvation on planarian microbial community composition, adult worms were starved for two weeks and then fed with either liver or boiled egg white once a week for eight consecutive weeks (Fig. 2A). Both liver and egg-white pieces were taken up by the worms, as indicated by the respectively dark-red and white colour of the worms immediately after feeding. The worms fed with liver responded differently than egg-fed and starved worms: their average total body surface increased over time, while the body surfaces of starved and egg-fed worms decreased (Fig. S2A). In addition, more than 80% of the liver-fed worms showed the process of binary fission, a natural way of asexual reproduction (Fig. S2B). For egg-fed worms this was 50%, starved animals did not show this behaviour.

Based on 16 S rRNA gene amplicon sequencing, the bacteria associated with egg- and liver-fed worms were studied to determine the effect of the diet on the associated communities. Two pieces of liver and two pieces of egg white were included to check if bacteria are present in the food source (Fig. S2C). Both liver pieces had very low read numbers, as well as 1 egg white (< 400). The second egg white piece had 3042 reads, while the samples in this experiment were represented by 36,540 reads on average (Fig. S2C). The reads present in the second egg white piece mainly corresponded to bacteria of the genera *Enhydrobacter* (21%) and *Methylobacterium* (14%) (Fig. S2D). In addition, 27% of the bacteria included genera that were present at 1–5% relative abundance (*e.g. Burkholderia* s.l., *Sphingobium and Paracoccus*) and 38% genera that were lower than 1% in relative abundance. The PCoA plot based on the weighted UniFrac distance showed a significant separation based on the time of feeding (*p-adj* = 9.99e^− 4^, *R*^2^ = 0.42), but not on food source (*p-adj* > 0.05, *R*^2^ = 0.02) (Fig. 2B). Heterogeneity of variance was observed (due to t_8_) along with a primary and secondary axis explaining respectively 42.0% and 18.0% of the total variation. At all time-points, Proteobacteria was the most abundant phylum, followed by Firmicutes and Bacteroidetes (Fig. S2E). No statistically significant differences between the different time points or food sources were found for the top 4 phyla. In the beginning of the experiment (t_0_), the phylum Proteobacteria was the most abundant in all samples (Fig. S2E). Medium that came into contact with the worms harboured similar bacteria communities, although in different abundances (Fig. 2C) compared to the worm samples. After 1, 3, 4 and 8 weeks of feeding, liver-fed worms harboured microbial communities with compositions similar to that of the corresponding egg-fed worms (Figs. 2C, S2E). However, when comparing the bacterial community compositions between the different weeks, we observed that worms at the starting point and worms fed for 1, 3 and 4 weeks had similar composition and diversity, but that worms fed for 8 weeks harboured communities with lower diversity, and unassigned genera of the Sphingomonadaceae family were relatively more abundant. Rarefaction curves are shown in Fig. S2F.

Within this section we conclude that an alternative diet does not induce major changes in the planarian microbiome, although it affects planarian physiology. The most notable changes were linked to temporal variations and unrelated to the diet (Table 2), indicating that (unknown) factors beyond our control also play a role in microbial variation. We address this further in the “Discussion” Section.

### External stressors influence the microbial composition

**Fig. 3:**
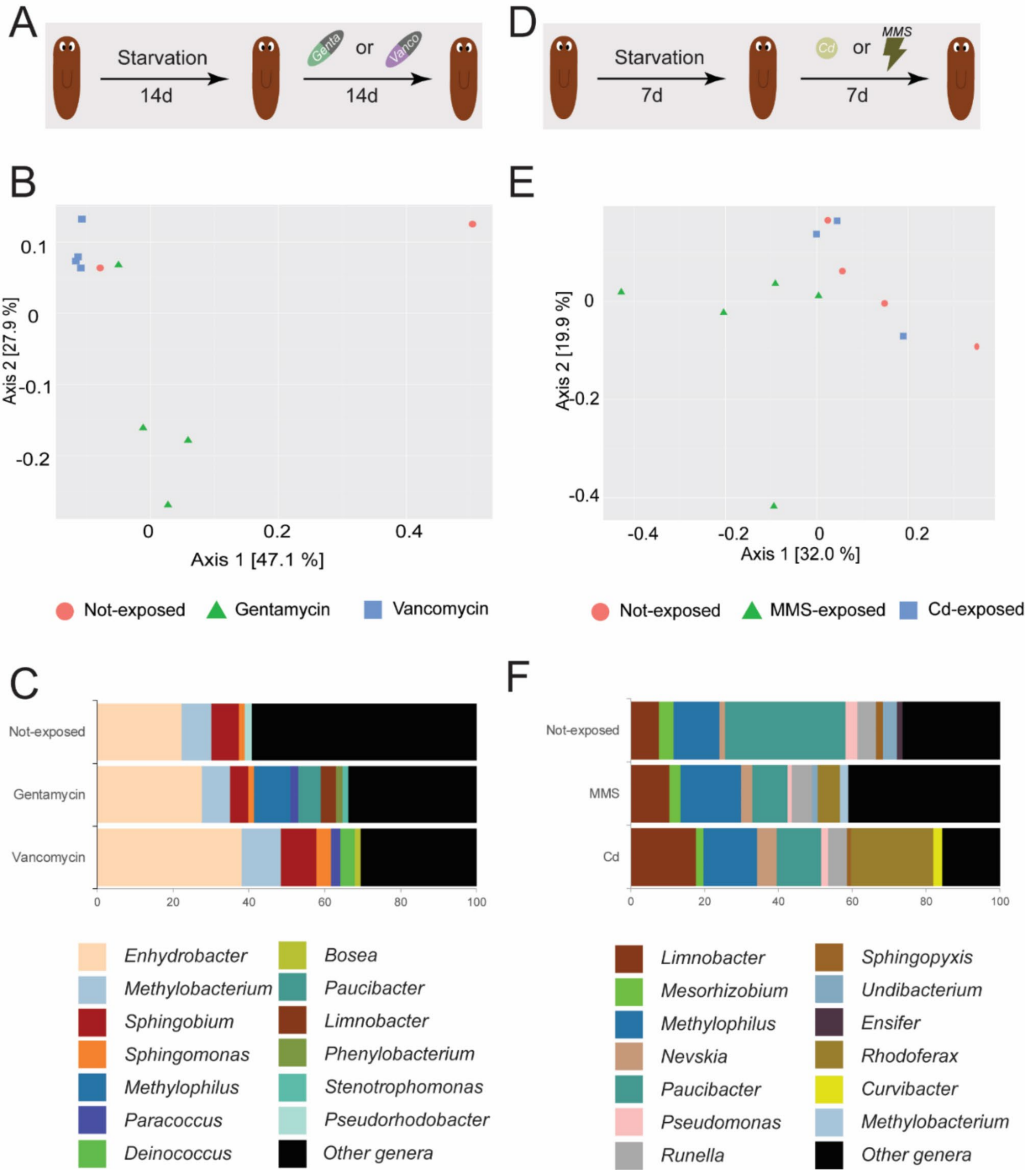
Microbial variation due to external exposures: antibiotics and chemical compounds. (**A**) Experimental setup for the *Data_Antibiotics* sequencing data. Adult worms were starved for 14 days and then exposed to gentamycin or vancomycin for 14 days (not-exposed *n* = 2, gentamycin *n* = 4, vancomycin *n* = 4). (**B**) PCoA of weighted UniFrac illustrating beta-diversity between not-exposed, gentamycin-exposed and vancomycin-exposed worms (*p-adj* = 9.99e^− 4^, *R*^2^ = 0.36, heterogeneity of variances). (**C**) Average relative abundances at the genus level for control and antibiotics exposed groups. Genera lower than 1.5% in RA were grouped in ‘other genera’. (**D**) Experimental setup for the *Data_Chemstress* sequencing data. Adult worms were starved for 7 days and then exposed to Cd or MMS for 7 days (not-exposed *n* = 4, Cd-exposed *n* = 3, MMS-exposed *n* = 4). (**E**) PCoA of weighted UniFrac illustrating beta-diversity between not-exposed, cadmium-exposed and MMS-exposed worms (*p* = 0.03, *R*^2^ = 0.26, homogeneity of variances). (**F**) Average relative abundances at the genus level for control and chemically exposed groups. Genera lower than 1% in RA were grouped in ‘other genera’. (RA: relative abundance, PCoA: principal coordinate analysis)

A previous study already indicated that the planarian microbiome is affected by exposure to silver nanoparticles (AgNP), an environmental relevant stressor^20^. Given the physiological responses to AgNP exposure, which were characterized by abnormal behaviour and decreased regenerative capacity^11^, a functional link between the planarian microbiome and physiology is hypothesized. Here, we further assess the effects of external stressors on the planarian microbiome. Therefore, we exposed worms to different compounds, including antibiotics and toxicants. In the first experiment we exposed worms to antibiotics that target gram-negative and gram-positive bacteria (Fig. 3A). Adult worms were starved and after exposure to gentamycin or vancomycin, samples were taken for 16 S rRNA gene amplicon sequencing. Beta-diversity analyses based on the weighted UniFrac distance, visualised in a PCoA plot, resulted in a statistically significant separation (*p-adj* = 9.99e^− 4^, *R*^2^ = 0.36) (Fig. 3B). However, due to heterogeneity of variance (*p-adj* = 9.99e^− 4^, *F* = 18.41), we cannot conclude that the effect was only due to antibiotic treatment. A pairwise comparison did not result in significant differences. For the three experimental conditions, the genera *Enhydrobacter*, *Methylobacterium*, *Sphingobium* and *Sphingmonas* were detected (Fig. 3C). The relative abundance of *Enhydrobacter* increased after gentamycin (more specifically, an increase by 5.3% was observed, from now on noted as + 5.3%) and vancomycin (+ 15.8%) exposure, compared to non-exposed worms. We observed a decrease in the abundance of *Sphingobium* (− 2.3%) after gentamycin exposure compared to non-exposed worms. In addition, an increase in the abundance of *Methylobacterium* (+ 2.5%), *Sphingobium* (+ 2.2%) and *Sphingomonas* (+ 2.3%) was observed after vancomycin exposure, compared to non-exposed worms. In non-exposed worms, *Pseudorhodobacter* was present, but not in the exposed groups. This was in contrast to bacteria of the *Paracoccus* genus that were detected in both exposed groups, but not in the non-exposed group. In gentamycin-exposed worms, *Methylophilus*, *Limnobacter*, *Paucibacter*, *Phenylobacterium* and *Stenotrophomonas* were detected, while *Bosea* and *Deinococcus* were present in the vancomycin-exposed worms. Differentially abundant genera are represented in Fig. S3A. Rarefaction curves are shown in Fig. S3C. Given the compositional nature of the data and the decreasing effect of the antibiotics treatment on the bacterial load, quantitative comparisons should be interpreted with caution.

In the second experiment we exposed adult worms to two toxicants, one DNA-alkylating agent (MMS), and a metal that alters cellular homeostasis by inducing oxidative stress (cadmium) (Fig. 3D). The effect of both agents on planarian physiology was already extensively studied^14,15,33^, allowing us to make links between physiological alterations and changes in microbiome composition. Beta-diversity analyses showed a statistically significant (*p-adj* = 0.03, *R*^2^ = 0.26) separation between exposure groups, however a pairwise comparison did not show significant differences (Fig. 3E). The most abundant genera in the three conditions included *Paucibacter*, *Runella*, *Methylophilus*, *Mesorhizobium*, *Limnobacter*, *Rhodoferax* and *Pseudomonas*. The relative abundance of *Runella* was comparable in the three conditions, while the abundance of other genera differed between the different treatments (Fig. 3F). The relative abundance of *Paucibacter* decreased 22.8% in MMS exposed worms and 20.5% in Cd-exposed worms, compared to non-exposed worms. For *Pseudomonas*, there was a decrease of 2.2% and 1.3% for the two treatments, respectively. Bacteria of the genus *Mesorhizobium* showed a decrease of 1.0 and 2.0% compared to the non-exposed worms. In contrast, the abundance of *Rhodoferax* increased, with 6.0% and 22.0% respectively in MMS-exposed worms and Cd-exposed worms compared to non-exposed worms. The abundance of *Limnobacter* increased 2.8% and 10.0% for MMS-exposed worms and Cd-exposed worms, respectively. For bacteria of the genera *Methylophilus* associated with MMS exposed worms, an increase of 4.0% was observed. For Cd exposed worms, this was an increase of 2.2% compared to non-exposed worms. A 1.3% and 3.8% increase in the abundance of *Nevskia* was observed for MMS exposed worms and Cd-exposed worms, compared to the non-exposed condition. Differentially abundant genera were detected as represented in Fig. S3B. Rarefaction curves are shown in Fig. S3D.

**Fig. 4:**
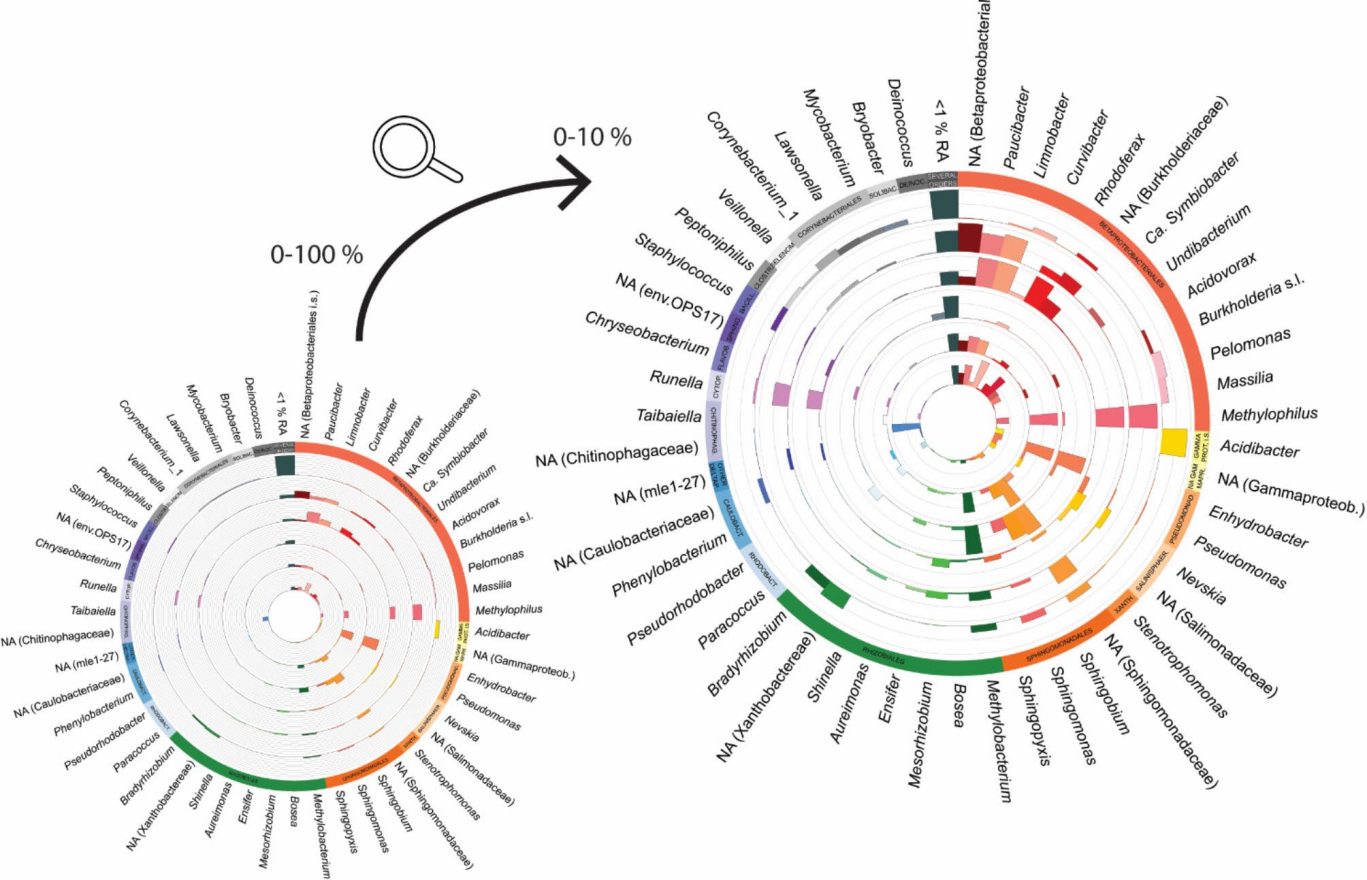
Patterns in the microbiome of *S. mediterranea* when exposed to external stressors. Circular plot showing the microbial composition after different types of chemical exposure in adult worms. From the inner to the outer circle: (1) not-exposed adult, (2) gentamycin exposed, (3) vancomycin exposed, (4) cadmium exposed, (5) MMS exposed and (6) silver nanoparticle exposed worms. The plots show the genera that are > 1% in relative abundance (coloured bars, labels next to outer circle), grouped by order (labels in coloured outer circle). The left plot shows the abundance from 0–100%, while the right plot shows 0–10%. The genera that have a RA above 10% are capped at 10% in the right plot. (not-exposed *n* = 16, Gentamycin *n* = 4, Vancomycin *n* = 4, cadmium *n* = 2, MMS *n* = 4, silver nanoparticle *n* = 12) (RA: relative abundance).

Knowing the effects of the individual compounds on the planarian microbiome, we aimed to understand if recurring microbial changes can be observed under environmental stress. In other words, we aim to investigate if specific bacterial taxa are more susceptible or resistant to stressors. For this, we made a larger comparison including the different types of exposures. We included the worms exposed to antibiotics, cadmium and MMS, and their corresponding controls. In addition, we also included data from our previous study examining the effects of AgNPs on planarians and their associated bacteria^20^. For each condition (controls, Cd, MMS, gentamycin, vancomycin and AgNPs) the average microbiome composition was calculated based on the relative abundances. Then each condition was plotted in a circular plot to allow comparisons (Fig. 4). Under the different stress conditions, we recurrently found that bacteria of the genera *Curvibacter*, *Ca. Symbiobacte*r, *Undibacterium* and *Acidovorax* were less abundant than in control worms. The abundances of *Paucibacter* and *Methylophilus* were low in vancomycin exposed worms and silver nanoparticle exposed worms compared to the other conditions.

For this section, we conclude that antibiotics, Cd and MMS induce changes in the microbiome composition (Table 2). Under these environmental stressors, several genera are recurrently affected, including *Curvibacter*, *Ca. Symbiobacte*r, *Undibacterium* and *Acidovorax*.

### The microbiome of *S. mediterranea* is variable over time and over different laboratories

**Fig. 5:**
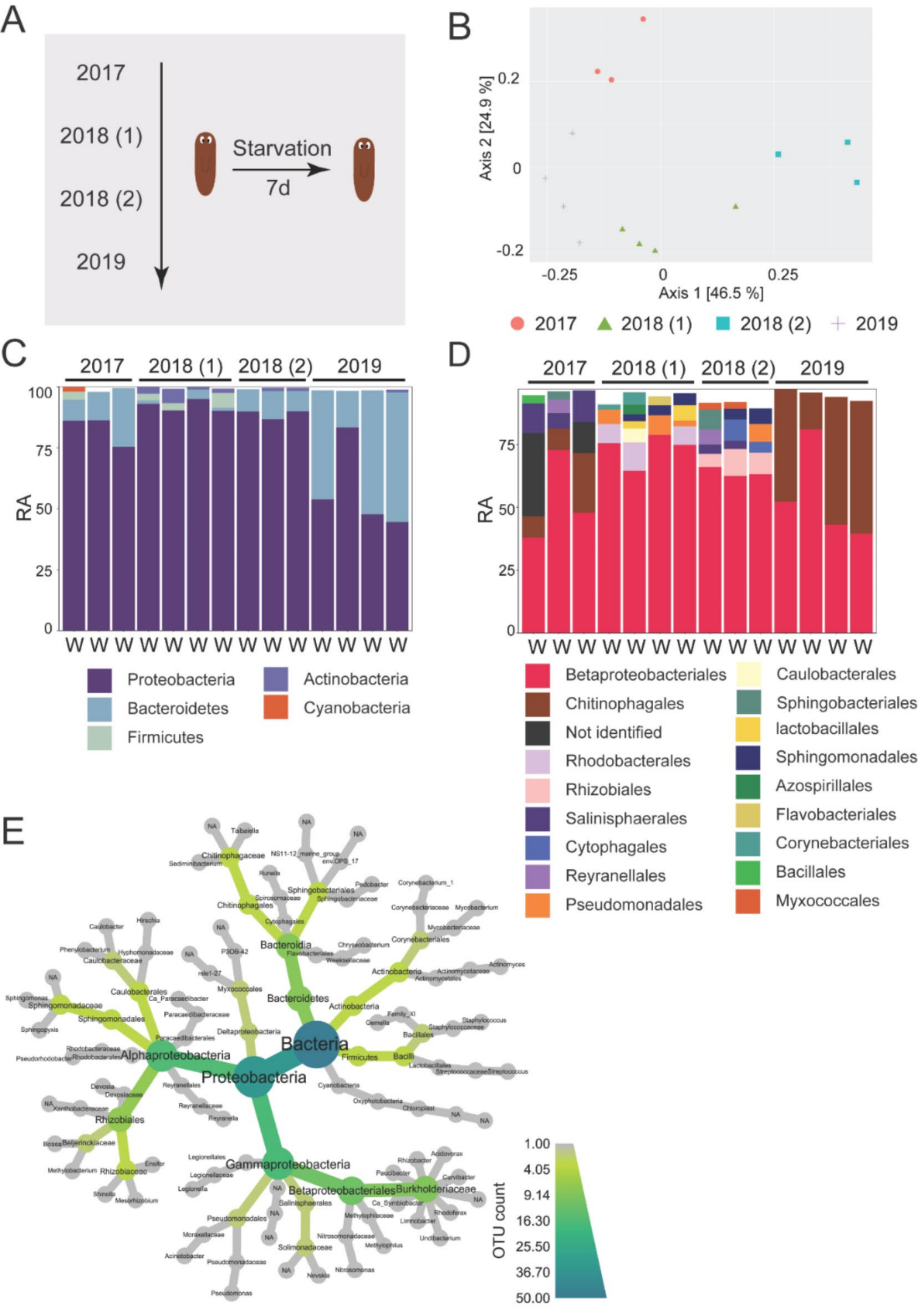
Temporal variation in the microbiome. (**A**) Experimental setup for the *Data_Time* sequencing data. Adult worms were starved for 7 days and then sampled for sequencing. This was done in 2017, 2018 and 2019. In 2018 two independent experiments were performed. (**B**) PCoA of weighted UniFrac illustrating beta-diversity between the different time-points (*p-adj* = 9.99e^− 4^, *R*^2^ = 0.73, homogeneity of variances). (**C**) Relative abundances (RA) at phylum level per individual for each time-point, filtered to remove taxa below 1% abundance. (**D**) Relative abundances (RA) at order level per individual for each time-point, filtered to remove taxa below 2% abundance. (**E**) Taxonomic relationships of the detected bacteria in the Data_Time sequencing data, node size and colour by abundance of the specific taxon. A darker color and larger node size means a higher abundance of this particular taxon on average in all samples included in the time analyses. (W: worm, RA: relative abundance, PCoA: principal coordinate analyses, 2017 *n* = 3, 2018 set 1 *n* = 4, 2018 set 2 *n* = 3, 2019 *n* = 4)

In our previous research, we noticed that physiological responses in planarians vary over time and in different laboratories. The planarian microbiome might be an explanatory variable in this context. In this section, we aim to report the variation present over time and over different laboratories, including factors that are beyond our control. First, to assess the variability of the planarian microbiome over time, we compared data of adult non-exposed worms from experiments performed in 2017, 2018 and 2019, sampled in the period from January to March (Fig. 5A). The PCoA plot based on the weighted UniFrac showed a significant separation based on the timing of the experiment (*p-adj* = 9.99e^− 4,^*R*^2^ = 0.73) (Fig. 5B). Homogeneity of variance was observed, along with a primary and secondary axis explaining respectively 46.5% and 24.9% of the total variation. Rarefaction curves are displayed in Fig. S4. On average, the most abundant phylum at all time-points was Proteobacteria, with a relative abundance (RA) of 83.4%, 92.9%, 89.1% and 50.8% for respectively 2017, 2018 (set 1), 2018 (set 2) and 2019 (Fig. 5C). The lower Proteobacteria content in 2019 was concurrent with a higher Bacteroidetes content (47.3% RA), compared to the other time points (1.5–13.3% RA) (Fig. 5C). The Firmicutes (2.7% RA) and Actinobacteria (2.8% RA) content was higher in set 1 of 2018 (Fig. 5C). At lower taxonomic levels, the microbiome was more variable (Fig. 5E). The most abundant order was Betaproteobacteriales for 2017, 2018 (set 1) and 2018 (set 2), with a RA of 49.7%, 75,4% and 64.5%. However, in 2019, bacteria of the order of Chitinophagales were the most abundant (47.1% RA) (Fig. 5D). The latter order was also detected in samples from 2017 (12.3% RA), together with Salinispaerales (10.5% RA) and several orders (19% RA) that could not be identified. In worms from 2018 (set 1), Rhodobacterales and Pseudomonadales were detected with a relative abundance of 7.4% and 5.6%, respectively (Fig. 5D). Samples from 2018 (set 2) appeared to harbour the most diverse bacterial communities, as Rhizobiales, Sphingobacteriales, Reyranellales, Cytophagales, Sphingomonadales, Salinispaerales, Myxococcales and Pseudomonadales were detected, in relative abundances ranging from 7.4 to 2.4% (Fig. 5D). As only 2 orders were detected at a relative abundance of 2% and more, bacterial communities from 2019 samples were less diverse than the other time points.

**Fig. 6:**
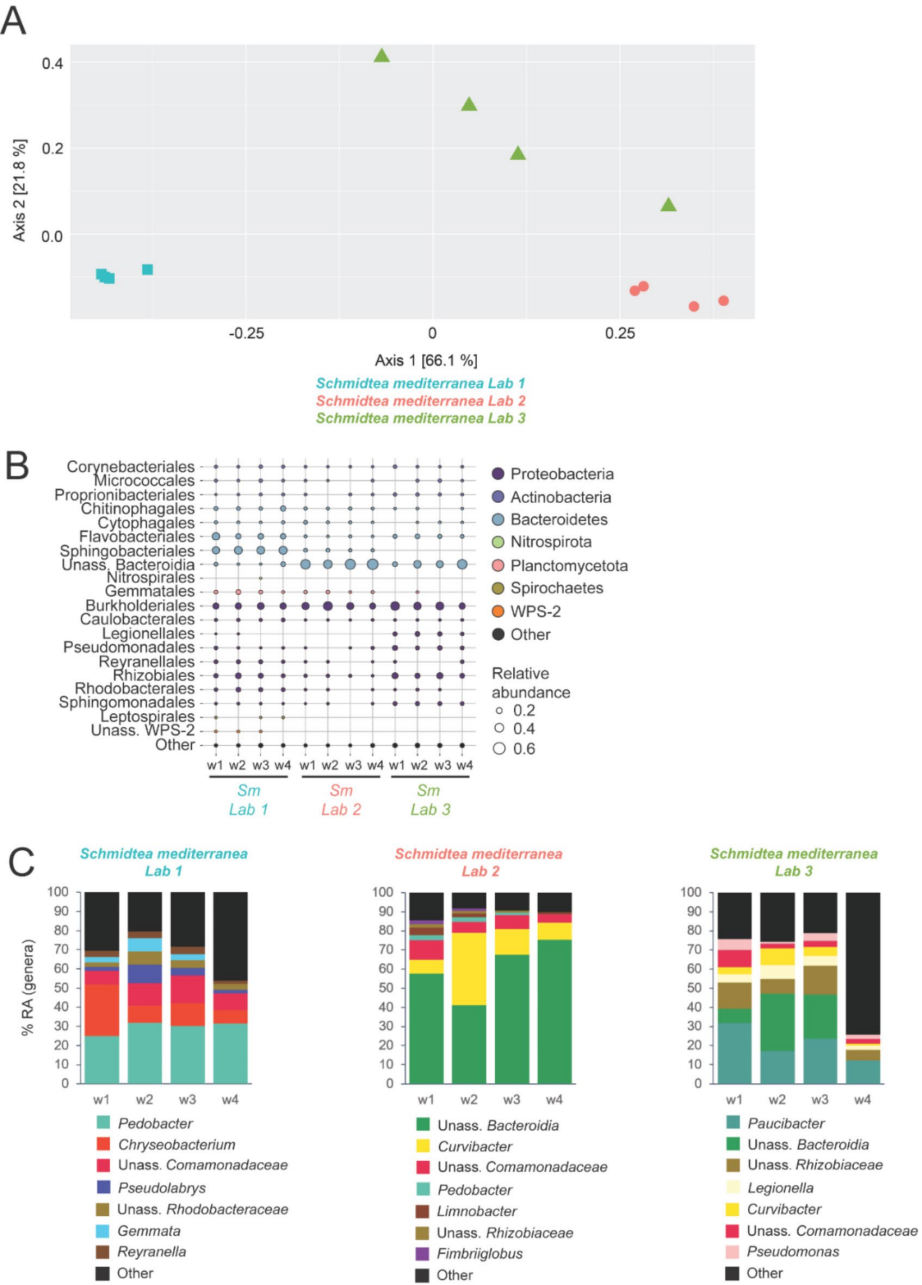
Diversity and composition of bacterial communities associated with *S. mediterranea* originating from different labs. (**A**) PCoA of weighted UniFrac illustrating beta-diversity between *S. mediterranea* originating from Lab 1, Lab 2 and Lab 3, based on *Data_Labs* (Adonis: ns, *R*^2^ = 0.800, homogeneity of variances). (**B**) Bubbleplot showing the relative abundances per sample, at bacterial order level for *S. mediterranea* originating from different labs. The bacterial orders are coloured by their corresponding phyla and the size of the bubble corresponds to the relative abundance. (**C**) Bar charts showing the top 7 bacterial genera with a RA > 1%. The group ‘Other’ contains genera with a RA > 1%, but not in the top 7. (PCoA: principal coordinate analyses, ns: not significant; w: worm, Sm: *S. mediterranea*, RA: relative abundance, Unass.: unassigned, Lab 1 *n* = 4, Lab 2 *n* = 4, Lab 3 *n* = 4)

Second, to assess variation between origins of planarians, we also included a comparison of the microbiomes of *S. mediterranea* that were collected from three different research laboratories. Historically, the origin of the strain used in most labs is located in the fountains of Montjuic (i.e. an artificial pond) in Barcelona, Spain^34^. Over the last decades, the animals were distributed among other research laboratories establishing their own planarian cultures, each with their preferences regarding food source, antibiotics use, light/day cycles etc. The PCoA plot based on the weighted UniFrac showed that the beta-diversity of bacteria associated with *S. mediterranea* originating from Lab 1, Lab 2 and Lab 3 was not statistically significantly different (Adonis: ns, *R*^2^ = 0.800) (Fig. 6A). A homogeneity of variance was observed, along with a primary and secondary axis explaining respectively 66.1% and 21.8% of the total variation. However, a pair-wise comparison showed a significant difference between the microbial beta-diversity of *S. mediterranea* originating from Lab 1 and Lab 2 (*p-adj* < 0.05, *R*^2^ = 0.869), Lab 1 and Lab 3 (*p-adj* < 0.05, *R*^2^ = 0.742) and Lab 2 and Lab 3 (*p-adj* < 0.05, *R*^2^ = 0.550). Rarefaction curves are displayed in Fig. S5C.

Bacteria belonging to Proteobacteria and Bacteroidetes dominate the microbiomes, although in different ratios depending on the specific samples (Fig. 6B). Samples originating from Lab 3 were dominated by Proteobacteria, while Lab 1 and Lab 2 showed a dominance of Bacteroidetes. Within the Proteobacteria, the ratio of Gammaproteobacteria and Alphaproteobacteria was in favour of the former for the specimens originating from all three lab environments. Within the Gammaproteobacteria, bacteria of the order Burkholderiales were the most dominant, while bacteria belonging to Rhizobiales were the most abundant Alphaproteobacteria (Fig. 6B). The phylum Bacteroidetes was mainly dominated by bacteria of the order Sphingobacteriales, Flavobacteriales and unassigned Bacteroidia (class Bacteroidia). Although we observed similarities at higher taxonomic levels, the majority of the top genera were different between the three lab environments (Figs. 6C, S5B). Several ASVs were differentially more or less abundant as shown in Fig. S5A.

In summary, we observed microbiome variation over time and variation over different laboratories (Table 2). At higher taxonomic levels, the majority of the conditions were dominated by Proteobacteria and Bacteroidetes. However, at lower taxonomic levels (i.e. starting from the order level), the microbiome became more variable. Given the central role of the microbiome in animal physiology, this observation might provide additional insight into the varying observations in planarian research (i.e. developmental and toxicological research), especially over time and over different laboratories.

### The microbiomes of closely related planarian species share bacterial taxa

**Fig. 7:**
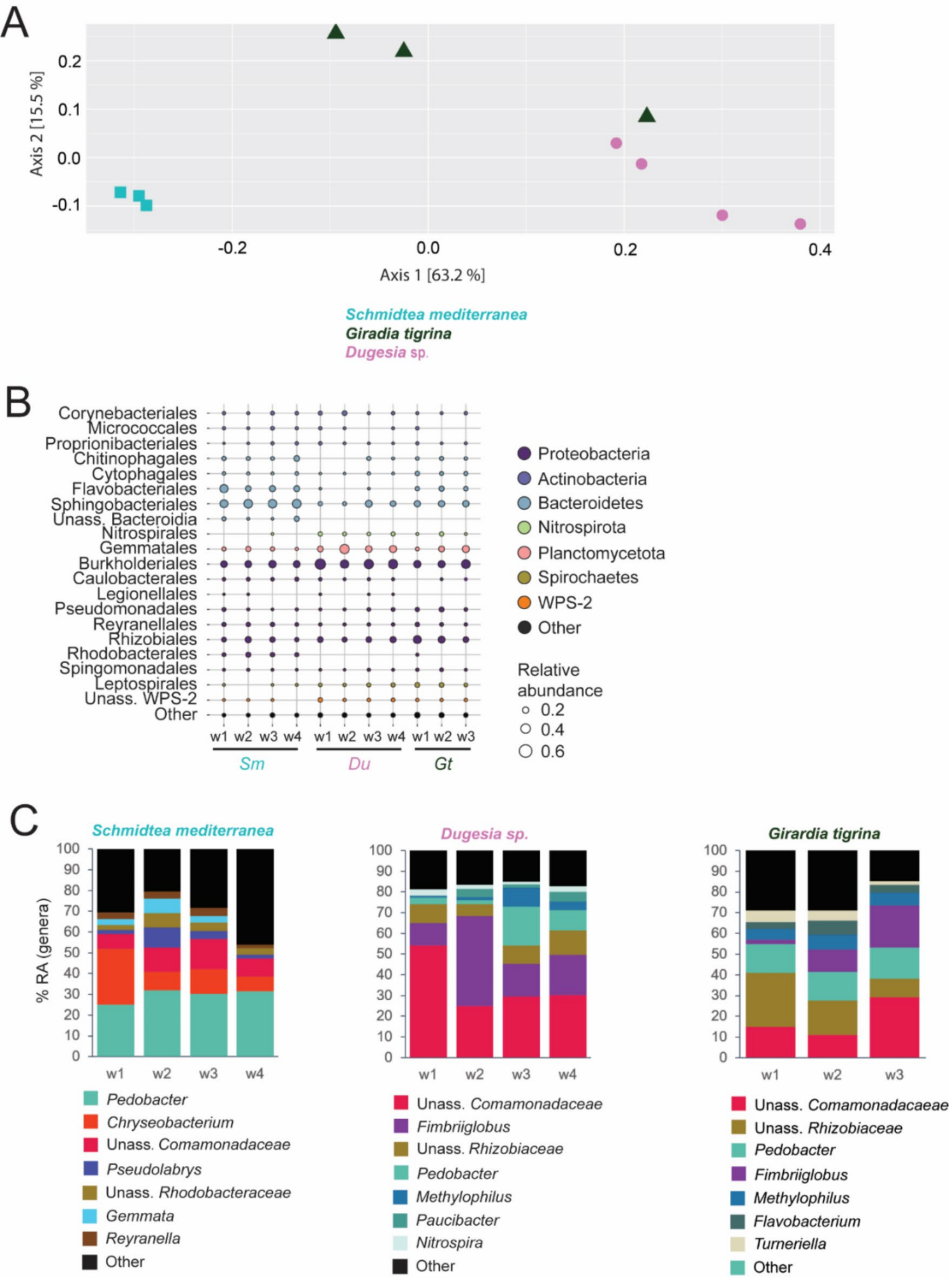
Diversity and composition of bacterial communities associated with lab-cultured *S. mediterranea*, *G. tigrina* and *Dugesia* sp. (**A**) PCoA of weighted UniFrac illustrating beta-diversity between *S. mediterranea*,* G. tigrina* and *Dugesia* sp. based on *Data_planarians* (Adonis: ns, *R*^2^ = 0.696, homogeneity of variances). (**B**) Bubbleplot showing the relative abundances per sample, at order level for the different planarian species. The orders are coloured by their corresponding phyla and the size of the bubble corresponds to the relative abundance. (**C**) Bar charts showing the top 7 genera with a RA > 1%, per worm species. The group ‘Other’ contains genera with a RA > 1%, but not in the top 7. (PCoA: principal coordinate analyses, ns: not significant; w: worm, Sm: *S. mediterranea*, Du: *Dugesia* sp. Gt: *G. tigrina*, RA: relative abundance, Unass.: unassigned, *Schmidtea mediterranea n* = 4, *Dugesia* sp. *n* = 4, *Girardia tigrina n* = 4)

In the context of phylosymbiosis, it is stated that animals closely related to each other have similar microbiomes as they evolved together^35^. In a first exploration for such a phylosymbiotic signal, we investigate if related planarian species share bacterial taxa. Therefore, we studied the bacterial diversity and composition associated with three different members of the planarian family of Dugesiidae, namely *S. mediterranea*, *G. tigrina* and *Dugesia* sp. Regarding beta-diversity, no statistically significant differences between the microbiomes of the three planarian species was observed (Adonis: ns, *R*^2^ = 0.696) (Fig. 7A). A homogeneity of variance was observed, along with a primary and secondary axis explaining respectively 63.2% and 15.5% of the total variation. However, a pair-wise comparison showed a significant difference between *S. mediterranea* and *G. tigrina* (*p-adj* < 0.05, *R*^2^ = 0.657), *S. mediterranea* and *Dugesia* sp. (*p-adj* < 0.05, *R*^2^ = 0.727) and *G. tigrina* and *Dugesia* sp. (*p-adj* < 0.05, *R*^2^ = 0.413). Rarefaction curves are displayed in Fig. S6C.

In general, 90% of the bacterial taxa of the microbiomes of the three planarian species belong to five bacterial phyla: Proteobacteria, Bacteroidetes, Planctomycetes, Spirochaetes and Actinobacteria (Fig. 7B). The top phylum in the microbiomes of *G. tigrina* and *Dugesia* sp. was Proteobacteria, while *S. mediterranea* was dominated by Bacteroidetes. The ratio of Gammaproteobacteria and Alphaproteobacteria was in favour of the former, with a dominance of Burkholderiales and Rhizobiales respectively (Fig. 7B). The phylum Bacteroidetes was mainly dominated by bacteria of the order Sphingobacteriales, Flavobacteriales and unassigned Bacteroidia (class Bacteroidia). Apart from the phyla Proteobacteria and Bacteroidetes, Planctomycetes, Spirochaetes and Actinobacteria were also present in the top 5 phyla (Fig. 7B). Compared to *S. mediterranea*,* G. tigrina* and *Dugesia* sp. showed a higher abundance of Planctomycetes and Spirochaetes. Bacteria of the order Gemmatales were the most abundant Planctomycetes, while Leptospirales were the most abundant Spirochaetes. The abundance of bacteria belonging to the phylum of Actinobacteria was similar over all samples. Within this phylum, bacteria belonging to the order of Corynebacteriales, Micrococcales and Propionibacteriales (class Actinobacteria) were the most abundant. At the lower taxonomic levels, several highly abundant genera were present in both *Dugesia* sp. and *G. tigrina*, including *Pedobacter*, *Fimbriiglobus*, *Methylophilus* and members of the unassigned Comamonadaceae and Rhizobiaceae (Figs. 7C, S6B). Differentially abundant genera are presented in Fig. S6A.

Based on the presented data, we conclude that closely related planarian species share bacterial taxa (Table 2). Including additional planarian species in the comparison, we confirm and extend the findings of Arnold et al. 2016^18^. The data point towards a phylosymbiotic signal, a potential future perspective for this study.

### The microbiome of *S. mediterranea* is highly variable, although a set of three genera is consistently present in adult animals

The combination of the different experiments and conditions gives us a unique opportunity to search for a set of stably associated bacteria in planarians. We hypothesize that such a set might have functional importance for planarians. However, when we consider all the different tested conditions, we could not find any genera that were present in all of the tested samples. This suggests that the planarian microbiome is highly variable and dependent on internal and external factors of variation.

Next, we aimed to find a recurring set of bacteria that is representative for a microbiome of a healthy adult, focussing on not-exposed animals. For this we compared control, adult animals of seven different experiments. First, per experiment, we selected the genera associated with non-exposed animals. Second, we calculated the average relative abundances of all genera over the included individuals. Third, we compared these to assess if common bacteria can be identified, while experimental and technical variations are present. We found a total of three genera that were in common for the seven experiments: *Curvibacter*, *Staphylococcus* and *Sphingomonas*. Additionally, in six of the independent experiments, *Methylophilus* was also found to be in common. In five of the independent experiments the genera *Paucibacter*, *Undibacterium*, *Pedobacter*, *Methylobacterium*, *Achromobacter*, *Streptococcus*, *Lawsonella*, *Pseudomonas*, *Acinetobacter* and *Rhodoferax* were also in common.

In summary, taking all experimental conditions of this study into account, no bacteria were found to be in common, indicating a variable nature of the planarian microbiome. However, restricting the analysis to a control condition, i.e. representing normal physiology (adult, non-exposed), we found a set of three genera that were recurrently present in all the associated experiments. We consider this set as relevant for future functional analyses and in the “Discussion” Section we hypothesize on specific functionalities for planarian physiology.

### The planarian microbiome might be important for the metabolism of biomolecules

Based on the results above, we aimed to gain insight into the functionality of the planarian microbiome. We used the already available 16 S rRNA sequences generated in this study, to predict bacterial functionality (Figs. S7–S8). Recent studies suggest a potential bias in the predictive value of analyses based on 16 S rRNA gene amplicons, as metagenomic and single-cell genomics have revealed significant within-species diversity^36^. Consequently, functional predictions based on 16 S data necessitate caution in interpretation. We present our predictive analysis findings as a preliminary investigation of potential hypotheses, acknowledging known limitations and refraining from definitive conclusions.

First, we focussed on the microbiome of control (not exposed) animals, to gain a deeper understand of the microbiome functions in an adult and regenerating animal. The principal component analysis showed that early stages of regeneration exhibit more variation than later stages of variation, with the primary and secondary axis explaining respectively 29.3% and 20.8% of the total variation (Fig. S7A). Given our hypothesis that the bacterial composition might be influenced by planarian physiology, and/or that bacterial metabolites influence the planarian host, we characterized the bacterial metabolism potential. Based on the KEGG metabolism categories the majority of predicted bacterial functionalities were categorized in amino acid metabolism, carbohydrate metabolism, energy metabolism, lipid metabolism and metabolism of cofactors and vitamins (Fig. S7B).

By comparing the predicted functionalities in adult vs. regenerating worms, we aim to screen for bacterial functionalities that are altered (and possibly of importance) in the regeneration process. Discriminatory features were found when comparing the functional microbiome features in adult worms vs. regenerating worms (Fig. S7C), as well as in the different time points during regeneration (Fig. S7D). Compared to regenerating worms, significant more features regarding signalling, carbohydrate and amino acid metabolism are present in adult worms (Fig. S7C). In contrast, in regenerating worms, predicted features correlated with carbohydrate and glycan metabolism were significantly enriched, as well a feature corresponding to a transporter (Fig. S7C). During the different time points; 0 dpa, 3 dpa, 7 dpa, 14 dpa and none (= adults), the observed discriminatory features were mainly linked to bacterial transporters and metabolism (i.e. fatty acid, carbohydrate and lipid metabolism) (Fig. S7D).

We performed an analogous analysis for worms that were exposed to environmental stressors in a first attempt to identify altered bacterial functions that might have an impact on planarian physiology. Given that we know the physiological responses after cadmium and MMS exposure, we focused on these two stressors. Based on the KEGG metabolism categories the majority of predicted bacterial functionalities were categorized in amino acid metabolism, carbohydrate metabolism, energy metabolism, lipid metabolism and metabolism of cofactors and vitamins (Fig. S8A). As only 2 replicates for the Cd-exposed worms are present, these were omitted in the further analysis, meaning that we focus the analysis on MMS-exposed worms. We compare the predicted functionalities between control and MMS-exposed worms (Fig. S8B). Although no features were statistically significant, the top 15 most discriminative features show that mainly ABC transporters are decreased in MMS-exposed worms, compared to the control. Alcohol dehydrogenase and alkanesulfonate monooxygenase, both linked to bacterial metabolism and members of the oxidoreductases are increased in the MMS-exposed worms.

### The planarian microbiome is present in the gut

So-far, we have studied the overall microbiome of planarians in different physiological conditions, without discriminating between different tissues. Future studies focussing on the bacterial identification in different locations might reveal further insights, aiding a more detailed understanding of their role in planarian physiology. As a starting point we localized bacteria in the planarian gut via FISH and SEM (Fig. S9).

## Discussion

Animals show variation in the complexity of their microbiome, as well as in the extent to which they depend on their associated bacteria^3^. Our study contributes to a better understanding of these host-microorganism associations in aquatic invertebrates, by comparing differences and commonalities within the microbiome of planarians, and correlating them to the physiology of planarians. This research is among the first to examine such a wide range of conditions.

We used the freshwater planarian *S. mediterranea*, to assess the composition, diversity and variation of its associated bacteria. *S. mediterranea* is a bottom dweller that predates or scavenges on aquatic arthropods, snails, oligochaetes and amphibian eggs^37^. Given their sensitivity to temperature and water quality changes, planarians are often used as an indicator of biodiversity^17^. Previous laboratory studies hypothesised the existence of functional dependencies, as shifts in the microbial community caused defects in host regeneration^18,19^. Most of the cultivable bacteria found in this study (Table S1) were previously detected in both lab-cultured and wild type *Schmidtea mediterranea*^18^, and in the close relative *Dugesia japonica*^19^. We found similar results via 16 S rRNA sequencing in *G. tigrina* as well (Figs. 7, S6). The shared bacterial genera include *Chryseobacterium*, *Pedobacter* and *Pseudomonas*. The genus *Chryseobacterium* (phylum Bacteroidetes) consists of gram-negative, rod-shaped bacteria that form yellow-orange coloured colonies on rich laboratory growth media, due to production of a flexirubin-type pigment^38^. Diverse bacteria of the *Chryseobacterium* genus have been found in freshwater habitats^39^, in the gut of freshwater copepods^40^ and in the mucus of fish^41^. *Chryseobacterium* KBW03 is endogenous in *D. japonica* and can produce indole, a bacterial metabolite that in high concentrations was found to inhibit planarian regeneration^19^. Kangale et al. 2020 also found the genus *Pedobacter* (phylum Bacteroidetes) in *S. mediterranea* and identified it as a new species called *Pedobacter schmidtea*^42^. These gram-negative, rod-shaped bacilli express a two-component system that reacts to external and internal stimuli such as changes in ion and gas concentrations, redox states, nutrient levels and cell density, although a direct link with the planarian physiology remains to be elucidated. *Pseudomonas* (phylum Proteobacteria) was already linked with the physiology of planarians, as an infection with a *Pseudomonas* strain resulted in impaired regeneration in *S. mediterranea*, with tissue lesions and degeneration^18^. Based on the conclusion of Arnold et al. (2016)^18^ and Lee et al. (2018)^19^ that imbalances in the planarian microbiome lead to regeneration defects, we further explored the composition and diversity of the planarian microbiome during different stages of regeneration. It is generally accepted that the microbiome is important for host growth and development through mechanisms including immune modulation, nutrient absorption and vitamin production^43^. The two independent experiments investigating the microbiome of regenerating animals showed little variation between experimental replicates and indicated that the microbiome of developing organisms undergo compositional shifts during the initial regeneration phases prior to stabilising to a pre-amputation state (Figs. 1C, D, S1A, B). More specifically, the ratio *Curvibacter* versus *Burkholderia* s.l. fluctuated during tissue regeneration (Fig. 1C, D). *Burkholderia* sensu lato (s.I.) (consisting of *Burkholderia Caballeronia* and *Paraburkholderia*) is a large and complex group, containing pathogenic, symbiotic and non-symbiotic strains from a range of environmental and clinical habitats^44^. The curved, rod-shaped gram-negative *Curvibacter* was previously isolated from aquatic environments^45^ and was also found in another study on *S. mediterranea*^18^. The genus was also identified as a dominant coloniser in *Hydra vulgaris*, a freshwater polyp (cnidarian) with regenerative capacity^31^. *Hydra* secretes a neuropeptide (NDA-1) that decreases the number of gram-positive bacteria in favour of *Curvibacter*, suggesting a specific selection for the bacterial genus by the host^46^. The quorum sensing mechanism of *Curvibacter* is modified by *Hydra*, preventing *Curvibacter* to express its flagella favouring host association^47^. It is possible that such interactions also occur in planarians. A planarian neuronal transcript with approximately 50% sequence similarity to NDA-1 was found in PlanMine -the planarian genome and transcriptome database^54^-, making it an interesting target for further investigation. *Hydra* without *Curvibacter* develop normally, but suffer from fungal infections that do not occur in normal cultures^31^. It suggests a protection against opportunistic pathogens, which is especially interesting during the regeneration stage in planarians, as at this stage the worms are particularly vulnerable for infections due to their wound site exposed to the outside world.

In addition to the observed shifts during tissue regeneration, we observed differences in the microbial composition in head and tail fragments, especially after 7 days of regeneration when head and tail segments harboured different bacterial communities (Fig. 1). Compared to vertebrates with well-defined body sites and cavities containing adapted bacterial taxa, the anatomy of *S. mediterranea* is considered relatively simple, with bacteria present in the intestines of the worm (Fig. S9). We observed bacteria in the larger primary intestinal branches (Fig. S9A) as well as in the deeper intestinal branches (Fig. S9B). Future studies focussing on both the location of the planarian microbiome and the intestinal cellular composition will provide more insight into potential tissue-specific locations of the detected species.

Many studies have shown that diet determines microbiome composition^32^ and is responsible for variation between individuals^48,49^. However, for *S. mediterranea* the influence of diet appeared to be limited when changing their food source from liver to egg-white (Figs. 2, S2E). This can be explained by the fact that both diets are rich in protein and that changes in macronutrients (carbohydrates, proteins and fats) in particular have an impact on the (human) gut microbiome^50^, but also by the fact that this experiment was not designed to fully differentiate between bacteria associated with the gut, epidermis and other structures. However, as shown by the decreased planarian body size (Fig S2A), and fewer successful fissions (Fig S2B), a diet composed of boiled egg whites is less suitable for *S. mediterranea* compared to liver. This can be attributed to the lower caloric value of egg-white (52 kcal per 100 g) compared to liver (165 kcal per 100 g). The strongest differences in microbial composition within the feeding experiments were observed when comparing feeding timepoints (Figs. 2C, S2E), suggesting that the microbiome of planarians microbiomes is not significantly affected by food or starvation, although there is temporal variation.

In contrast, external toxicants had a strong effect on the microbial community composition of planarians. Both the heavy metal cadmium and the carcinogenic alkylating agent MMS lowered the abundance of Firmicutes and Gammaproteobacteria with a concomitant increase in Bacteroidetes (Fig. 3F), which was consistent with reports in rodents^51^. While the specific links between the changing microbiome and physiological responses remain to be elucidated, detailed descriptions of the induced physiological responses are already available for most of the tested compounds. Exposure to Cd induces neurotoxicity in freshwater planarians, characterized by abnormal locomotion and behaviour^52^. MMS is known to induce DNA damage in planarians, resulting in a decrease in stem cell proliferation and differentiation, leading to impaired regenerative success^15^. In addition, in this study, we predicted microbiome functionality and although not statistically significant, we found a trend indicating that ABC transporters are decreased in MMS-exposed worms, compared to controls and that specific oxidoreductases are increased (Fig. S8B). Increasing the sample size in a future study will advance our understanding of this response. In our previous study on AgNPs, we showed that mainly Betaproteobacteriales were affected and that the microbiome of AgNP-exposed worms was enriched in genes related to the fatty acid metabolism. Given that AgNPs are linked with oxidative stress and are able to damage membranes, we hypothesise that bacteria efficient in degrading damaged membranes and synthesizing new fatty acids to rebuild the membranes are more likely to tolerate AgNP exposure. It is already known that AgNPs induce neurodevelopmental toxicity and abnormal behaviour in planarians, characterized by stem cell alternations^11^. However, the causal relationship between the bacterial changes and the elicited neurotoxic effects has yet to be fully established. The broad-spectrum antibiotics gentamycin and vancomycin had limited effects on gram-positive or gram-negative bacteria (Fig. 3C). Concentrations were based on literature, but it is possible that these concentrations were too low, that treatment times were inadequate, that uptake was limited by the planarian protective mucus layer, or because of bacterial resistance to the antibiotics. To investigate whether specific bacteria are consistently more affected in stressed conditions, we conducted a comparative analysis of the various external stressors tested. This investigation also incorporates data from our previous study detailing the effects of exposure to silver nanoparticles on the microbiome^20^. Overall, we found that the genera *Curvibacter*, *Ca. Symbiobacte*r, *Undibacterium* and *Acidovorax* were consistently less abundant under stress conditions than in control worms (Fig. 5). While the reasons for the sensitivity of these specific groups remain unknown, the compounds involved exhibit a diverse range of modes of action and mechanisms of toxicity. Understanding these dynamics, alongside the physiological implications for the planarians, highlights the potential of using microbiomes as biomarkers for assessing chemical toxicity.

The variety of experimental setups employed in this study enable us to assess the susceptibility and importance of specific bacterial groups, and explore whether planarians have a consistent set of bacteria within their microbiome. After comparing all tested conditions, we were not able to identify common genera. However, when comparing adult control animals, representing a healthy microbiome, we found that *Curvibacter*, *Paucibacter*, *Pedobacter*, *Pseudomonas* and *Rhodoferax* were, among others, consistently present. These genera were also identified in previous studies on planarian microbiomes^18,19^, and may have a function in planarian physiology, such as biomolecule metabolism (Fig. S7). While we recognize the need for future research employing metagenomic and metatranscriptomic approaches to further elucidate the functional dependence of planarians on their microbiomes, our findings already provide valuable insights. Specifically, we observed a decrease in *Curvibacter* in response to external stressors, suggesting an interaction between planarian physiology and microbial composition, particularly considering its fluctuations during regeneration. Yet other genera, such as *Ca. Symbiobacte*r, *Undibacterium* and *Acidovorax* were less abundant under stress conditions, implying that other processes are also taking place. Overall, *Curvibacter* emerges as an interesting target for further investigation due to its presence in healthy individuals, its responsiveness to environmental stress and its fluctuations during regeneration. Furthermore, given its anti-fungal properties observed in regenerative *Hydra*^31^, we hypothesize that *Curvibacter* may have a functional role in planarian physiology. Our data highlight the interactions between the planarian host, its microbiome and the environment. A limited number of environmental samples restricted our ability to fully assess the competition and influence of the environmental microbiome, which is a limitation of the study. However, the experimental replicates and multiple conditions in this study provide a comprehensive comparison, offering insights that extend beyond previous research. We also observed temporal microbial community variation between experiments (Fig. 5), despite the controlled laboratory environment (constant temperature, darkness, consistent feeding pattern). In addition, we observed variation between individual samples within a single experiment (e.g. Figure 1C-D) and between laboratories (Fig. 6). In humans this is often attributed to host genetics^53^, and while the planarians used in this study are assumed to have identical genetic backgrounds, additional experiments are required to explore this possible path.

## Conclusion

In this study, we characterised the microbiome of *Schmidtea mediterranea* in detail and found that it is dominated by Betaproteobacteriales (Phylum Proteobacteria) and is likely present in the planarian gut. Our innovative approach, incorporating diverse experimental setups and independent replicates, enabled us to be the first to compare such a wide range of conditions. We documented the variability present in the planarian microbiome under standard experimental circumstances – a critical, but often underreported aspect for understanding host-microorganism interactions. Key factors driving bacterial composition and diversity were identified, including the regenerative stage, which significantly altered the planarian microbiome. We hypothesize that factors produced during the regeneration process favour a different bacterial composition. External exposures also caused shifts in the bacterial composition, with specific taxa recurrently being affected by different types of stressors. Moreover, we identified key genera that consistently appeared in healthy adult animals, such as *Curvibacter*, suggesting potential functional significance in the health and physiology of *S. mediterranea*. Our study highlights that not all variations can be controlled or explained, emphasizing the need for diverse conditions and replicates in microbiome research to capture host-microorganism dynamics. By addressing the variability of the planarian microbiome, our work serves as a starting point towards exploring the functional relationships between planarians and their microbial partners.

## Electronic supplementary material

Below is the link to the electronic supplementary material.

Supplementary Material 1

Supplementary Material 2

## Data Availability

The sequencing datasets generated and/or analysed during the current study are available in the Sequence Read Archive (SRA) repository, accessible via PRJNA1036534 and PRJNA675880. Additional data is available from the corresponding author on reasonable request.
