# Supplementary material for: Impact of host physiology and external stressors on the bacterial community of Schmidtea mediterranea
Source: Sci Rep. 2025 Feb 5;15:4398. doi: 10.1038/s41598-025-86920-0 (PMC11799148; doi:10.1038/s41598-025-86920-0)
Supplement: Supplementary file 2 — Supplementary Material 2 [file 41598_2025_86920_MOESM2_ESM.docx]

Supplementary Material

# 1 Supplementary data

The resulting ASV tables with taxonomy and mapping file can be found in the supplied excel file.

Supplementary Material

# 2 Supplementary materials and methods

#### High-throughput sequencing

#### DNA extraction

Frozen planarian tissue was thoroughly homogenised in 100 µl of homogenization buffer (181 mM NaPO_4_, 121 mM guanidine isothiocyanate, pH 7.5) by vortexing until no tissue clumps remained visible. This homogenate was used to extract the total DNA. For this, 10 µl lysis solution (150 mM NaCl, 4% SDS, 0.5 M TRIS, pH 7.5) and 32 μl inhibitor removal solution (120 mM aluminium ammonium sulphate dodecahydrate, pH 3.2) was added, followed by vortexing. Next, an equal volume (140 μl) of phenol:chloroform:isoamyl alcohol, pH 6.5–8.0 (Invitrogen, cat. id 15593031), was added, and the mixture was thoroughly vortexed, after which samples were centrifuged at maximum speed (18 000 × g) for 2 min. The upper aqueous phase containing the DNA was recovered, and after addition of 60 μl of protein precipitation solution (5 M NaCl, 22 mM citric acid anhydrous salt, 29 mM trisodium citrate dehydrate, pH 5.0–5.2) the samples were incubated on ice for 10 min. After centrifugation, the pellet-free supernatants were used for the next step. Per 100 µl supernatant, 160 µl DNA-precipitation solution was added (5 M guanidine hydrochloride, 30 mM TRIS, 9 % isopropanol, pH 6.8), well mixed, and loaded onto a Silica mini-spin column (Epoch Life Science, catalogue-id 1920-050). By centrifuging 1 min at maximum speed (18 000 × g), DNA was bound to the column. Next, the column was washed twice with 200 µl ethanol-based wash solution (10 mM Tris, 100 mM NaCl, 50 % EtOH, pH 7.5) and dried by an additional centrifugation at maximum speed (18 000 × g) for 3 min. Finally, the DNA was eluted in 25 µl of 70° C pre-warmed elution buffer (10 mM TRIS, pH 8.0) and all samples were stored at -20° C. All solutions were made with molecular biology-grade products, diluted with molecular biology grade water, filter-sterilised before use and used within two months. Extraction blanks were included in the sequencing runs and analyses.

#### Library preparation for Ion Torrent sequencing

The Ion Torrent library preparation consisted of a nested primer approach. In the first round of PCR, 341F (5′-TAC GGG AGG CAG CAG-3’) and 806R (5′-GGA CTA CVS GGG TAT CTA AT-3’) primers were used. In the second-round of PCR, sequencing adaptor Ion Xpress barcoded 341F (5′-CCA TCT CAT CCC TGC GTG TCT CCG ACT CAG CTA AGG TAA CGA TTA CGG GAG GCA GCA G-3’) with P1 adapted 806R (5′-CCA CTA CGC CTC CGC TTT CCT CTC TAT GGG CAG TCG GTG ATG GAC TAC VSG GGT ATC TAA T-3’) primers were used.

Per sample, a reaction mixture of 20 µl was prepared, containing 1 µl extracted DNA, 1 × GoTaq Master Mix (Promega Corporation), 0.5 µM forward primer and 0.5 µM reverse primer. The PCR program for the first round of amplicon generation started with an initial denaturation for 4 min at 95° C, followed by a 30 sec denaturation at 95° C, a 45 sec annealing at 53.4° C and a 2 min extension at 72° C, the latter steps were repeated for a total of 25 cycles. The reaction was ended by a final 5 min extension at 72° C. For generating barcode adapted amplicons (second round), the same thermocycler program was used, although with an annealing temperature of 65° C. After each round of PCR, the amplified DNA was purified using the AMPure XP beads (Beckman Coulter), visualised on agarose gels, and quantified using a Quant-iT dsDNA BR Assay Kit (ThermoFisher Scientific, Burlington, ON, Canada) on a Qubit 2.0 Fluorometer (ThermoFisher Scientific, Burlington, ON, Canada). Template-free and positive controls were included.

The barcoded amplicons were pooled in equimolar amounts and the library dilution factor was determined using an Ion Library Quantitation Kit and a QuantStudio 3 system. Sequencing was done on an Ion S5 XL (Life Technologies Inc., Carlsbad, CA) running Torrent Suite 5.10.0, on an Ion 530 chip using 400 bp chemistry. An Ion 520 & Ion 530 Kit-Chef on an Ion Chef system was used to prepare DNA for sequencing.

#### Library preparation for Illumina sequencing

For Illumina sequencing, a library was prepared using two rounds of PCR. In the first round of PCR, an amplicon of 444 bp was generated using primers 341F and 785R (Klindworth et al. 2013), with an Illumina adapter overhang nucleotide sequence adaptor: 5’-TCG TCG GCA GCG TCA GAT GTG TAT AAG AGA CAG CCT ACG GGN GGC WGC AG-3’ and 785R-adaptor: 5’-GTC TCG TGG GCT CGG AGA TGT GTA TAA GAG ACA GGA CTA CHV GGG TAT CTA ATC C-3’. After the first round of PCR amplicons were cleaned up using the AMPure XP kit according to manufacturer’s instructions. In the second round of PCR, the Illumina Nextera XT indices (Set A, FC-131-2001, and D, FC-131-2004) were attached.

A reaction volume of 25 µl per sample was prepared containing 2 µl extracted DNA, 1 × Reaction Buffer with 1.8 mM MgCl_2_, 200 µM dNTP mix, 5 % DMSO, 200 nM forward primer, 200 nM reverse primer and 1.2 U Enzyme Blend (FastStart High Fidelity PCR system, dNTPack (Roche)). The PCR program started with an initial denaturation for 2 min at 95° C, followed by a 30 sec denaturation at 95° C, a 30 sec annealing at 53° C and a 1 min extension at 72° C, the latter steps were repeated for a total of 35 cycles. The reaction was ended by a final 7 min extension at 72° C. AMPure XP beads (Beckman Coulter) were used to purify the resulting amplicon. Thereafter 20 µl of beads were gently mixed with 25 µl PCR product, followed by 5 min incubation at room temperature. Using a magnetic stand, the supernatant was cleared and removed and the remaining beads were washed twice with 200 µl 80 % ethanol. The beads were air-dried and gently mixed with 27.5 µl of 10 mM Tris pH 8.5 elution buffer, followed by 10 min incubation at 37° C. After clearing the supernatant using the magnetic stand, 25 µl of the supernatant was transferred to a new sample container. Using the Nextera XT Index Kit v2 Set A (FC-131-2001) and D (FC-131-2004), unique index combinations for each sample were selected. 2 µl of the purified PCR product was used as input in the second index PCR, in a reaction volume of 25 µl per sample containing 1 × Reaction Buffer with 1.8 mM MgCl_2_, 200 µM dNTP mix, 5 % DMSO, 200 nM forward primer (Nextera XT Index Kit), 200 nM reverse primer (Nextera XT Index Kit) and 1.2 U Enzyme Blend (FastStart High Fidelity PCR system, dNTPack (Roche)). The PCR program used was the same as in the first round, except only for 25 cycles. The PCR purification was also performed using the AMPure magnetic beads. Template-free and positive controls were included.

After cleanup, the DNA concentrations of the resulting amplicons were quantified using the Qubit dsDNA HS assay kit (Invitrogen) and the Qubit 2.0 Fluorometer (Invitrogen) prior to pooling amplicons in equimolar concentrations in 100 μl TE buffer, resulting in a final library of 4 nM. Correct amplicon size and integrity were checked by analysing 1 μl of the library on a DNA-chip (DNA 1000 kit, Agilent Technologies) on a 2100 Bioanalyzer (Agilent) according to the manufacturer's instructions. The resulting quality checked amplicon pool was sequenced using a MiSeq Reagent Kit v3 (600 cycle) (MS-102-3003) and 15 % PhiX spike-in (FC-110-3001) on a MiSeq sequencing system (Hasselt University, Belgium), following the manufacturer’s protocol.

Bioinformatics processing

Reads were demultiplexed using the build-in platform software. Resulting FASTQ files were analysed in RStudio 1.2.1335, R version 3.5.3 using the DADA2 (version 1.10.1) microbiome pipeline to infer exact amplicon sequence variants (ASVs) from raw sequencing data (Callahan et al. 2016). For Ion Torrent single-end reads, the DADA2 script was adapted with custom filtering and trimming parameters: trimLeft=22, truncLen=c(230), maxN=0, maxEE=c(2), truncQ=2, rm.phyx=FALSE. For Illumina paired-end reads, the sequences were filtered and trimmed using the following parameters: trimLeft=c(17,21), truncLen=c(290,280), maxN=0, maxEE=c(2,2), truncQ=2, rm.phix=TRUE. Error rates were inferred and the filtered reads were de-replicated and de-noised using the DADA2 default parameters. After merging paired reads (Illumina only) and removal of chimeras, an ASV table was built and taxonomy was assigned using IdTaxa (DECIPHER, version 2.10.2) (Murali et al. 2018) and the SILVA_SSU_r132_March2018.RData training set (Quast et al. 2013; Yilmaz et al. 2014). The resulting ASVs and taxonomy tables were combined with the metadata file into a physeq object (phyloseq, version 1.26.1) (McMurdie and Holmes 2013) and a tree was generated using rtree (ape, version 5.3) (Paradis and Schliep 2019).

To assess DNA contamination, we included extraction blanks and non-template controls for each processing batch. After sequencing, detected contaminants were removed from the dataset using the package Decontam (version 1.2.1) applying the prevalence method with a 0.5 threshold value (Davis et al. 2018). Organelle sequences such as mitochondria and chloroplasts were also removed. In addition, a manual screen for suspicious reads was performed, to remove possible contaminants present in blanks. Of note, in one non-exposed sample of the dataset *Data_Chemstress*, the abundances of the genera *Agathobacter*, *Blautia* and *Faecalibacterium* were aberrant compared to other samples. Since these bacteria are commonly found in the human gut (Alvarez-Mercado et al. 2019), we omitted this sample from the analyses due to suspicion of sample contamination. In other samples within this experiment, and in other experiments of this study, these genera were not present or were detected in very low abundances. In addition, environmental controls (e.g. planarian medium, food…) were included in the analyses and reported when available.

Taxonomic analyses

For downstream taxonomic analyses subsets per experiment were made and further analysed in RStudio 1.2.1335, R version 3.5.3 on Windows 10 64-bit after removing samples with low reads (< 1000). Relative abundances were visualised per taxon in bar charts (ggplot2, version 3.1.1 (Wickham 2009) or Excel 2016) or circos plots (Circos software, version 0.69–6). Bubble plots and tree-based visualizations showing hierarchy of the taxa were made using ggplot2 and metacoder (version 0.3.3) (Foster et al. 2017), respectively. Venn diagrams were constructed using the freely accessible bioinformatics tools of UGent (UGent 2020). Statistical differences in the mean taxon relative abundance between groups were calculated by the Wilcoxon Rank Sum test. Observed ASV count, Simpson’s and Shannon’s diversity indices were calculated as alpha-diversity metrics using the vegan package (version 2.5.4) (Oksanen et al. 2016). Simpson’s index is reported as *1-D,* where *D=sump_i_^2^*, while Shannon’s index is defined as *H= -sump_i_log b p_i_* where *p_i_* is the proportional abundance of species *i* and *b* the base of the logarithm. The results were summarized in boxplots. Analysis of variance (ANOVA) and the Tukey Honest Significant Differences method (Tukey HSD) were used for hypothesis testing. When assumptions of normality and homoscedasticity were not met after transformation, a Kruskal-Wallis Rank Sum test and a Wilcoxon Rank Sum test was performed. A normal distribution was assessed using the Shapiro-Wilk test, while homoscedasticity was evaluated using the Bartlett test. For beta-diversity the weighted UniFrac (Lozupone et al. 2011) was calculated (vegan) and visualised using principal coordinate analyses (PCoA). The Adonis function (vegan, permutational manova) was used for hypothesis testing, and the Betadisper function (vegan) was used to assess homogeneity of variances. Differential abundance testing was done by the DESeq2 algorithm (version 1.22.2) with parameter fitType=”local” (Love et al. 2014) and the results were visualised using a lollipop chart (Excel 2016) or volcano plot via the R-package EnhancedVolcano (Blighe et al. 2018). When multiple experiments were analysed and visualized together (*e.g.* temporal variation, circos plots, Venn-diagrams), the data were aggregated at genus level, summing aggregating features. All performed statistical tests were corrected for multiple testing using false discovery rate.

Functional prediction

Functional prediction was done using MicrobiomeAnalyst (Lu et al., 2023, Chong et al., 2020, Dhariwal et al., 2017). The ASV and taxonomy tables, together with the metadata were used to predict functional profiles using Tax4Fun2. The resulting output was used for functional profiling, more specifically to generate a diversity overview showing barcharts of the relative abundances of the features (grouped by KEGG metabolism categories). A clustering analysis was done using a heatmap and principal component analysis. A linear discriminant analysis effect size (LEfSe) was performed using the parameters p-value cutoff: 0.1 (FDR-adjusted) and Log LDA score > 2.0. The resulting features were further identified using the KEGG orthology database (Kanehisa & Goto, 2000, Kanehisa, 2019, Kanehisa et al., 2023).

#### Community fingerprinting

As input for the PCR, 2 µl DNA was mixed with 23 µl mastermix containing 1x DreamTaq buffer (ThermoScientific), 40 µM dNTPs (Promega), 200 µM forward primer (ITSF: 5′-GTCGTAACAAGGTAGCCGTA-3′), 200 µM reverse primer (ITSReub: 5′-GCCAAGGCATCCACC-3′) and 1 U DreamTaq polymerase (ThermoScientific). The PCR program started with an initial denaturation for 3 min at 95° C, followed by a 30 sec denaturation at 94° C, a 1 min annealing at 55° C and a 1 min extension at 72° C, the latter steps were repeated for a total of 35 cycles. The reaction was ended by a final 7 min extension at 72° C.

#### Additional references

Álvarez-Mercado AI, Navarro-Oliveros M, Robles-Sánchez C, Plaza-Díaz J, Sáez-Lara MJ, Muñoz-Quezada S, Fontana L, Abadía-Molina F. Microbial Population Changes and Their Relationship with Human Health and Disease. Microorganisms. 2019 Mar 3;7(3):68. doi: 10.3390/microorganisms7030068. PMID: 30832423; PMCID: PMC6463060.

Blighe, K, S Rana, and M Lewis. 2018. “EnhancedVolcano: Publication-ready volcano plots with enhanced colouring and labeling.” https://github.com/kevinblighe/EnhancedVolcano.

Callahan BJ, McMurdie PJ, Rosen MJ, Han AW, Johnson AJ, Holmes SP (2016) DADA2: High-resolution sample inference from Illumina amplicon data. Nat Methods 13(7):581-3 doi:10.1038/nmeth.3869

Chong J, Liu P, Zhou G, Xia J. Using MicrobiomeAnalyst for comprehensive statistical, functional, and meta-analysis of microbiome data. Nat Protoc. 2020 Mar;15(3):799-821. doi: 10.1038/s41596-019-0264-1. Epub 2020 Jan 15. PMID: 31942082.

Davis NM, Proctor DM, Holmes SP, Relman DA, Callahan BJ (2018) Simple statistical identification and removal of contaminant sequences in marker-gene and metagenomics data. Microbiome 6(1):226 doi:10.1186/s40168-018-0605-2

Dhariwal A, Chong J, Habib S, King IL, Agellon LB, Xia J. MicrobiomeAnalyst: a web-based tool for comprehensive statistical, visual and meta-analysis of microbiome data. Nucleic Acids Res. 2017 Jul 3;45(W1):W180-W188. doi: 10.1093/nar/gkx295. PMID: 28449106; PMCID: PMC5570177.

Foster ZS, Sharpton TJ, Grunwald NJ (2017) Metacoder: An R package for visualization and manipulation of community taxonomic diversity data. PLoS Comput Biol 13(2):e1005404 doi:10.1371/journal.pcbi.1005404

Kanehisa M, Furumichi M, Tanabe M, Sato Y, Morishima K. KEGG: new perspectives on genomes, pathways, diseases and drugs. Nucleic Acids Res. 2017 Jan 4;45(D1):D353-D361. doi: 10.1093/nar/gkw1092. Epub 2016 Nov 28. PMID: 27899662; PMCID: PMC5210567.

Kanehisa M, Furumichi M, Sato Y, Kawashima M, Ishiguro-Watanabe M. KEGG for taxonomy-based analysis of pathways and genomes. Nucleic Acids Res. 2023 Jan 6;51(D1):D587-D592. doi: 10.1093/nar/gkac963. PMID: 36300620; PMCID: PMC9825424.

Kanehisa M, Goto S. KEGG: kyoto encyclopedia of genes and genomes. Nucleic Acids Res. 2000 Jan 1;28(1):27-30. doi: 10.1093/nar/28.1.27. PMID: 10592173; PMCID: PMC102409.

Klindworth A, Pruesse E, Schweer T, et al. (2013) Evaluation of general 16S ribosomal RNA gene PCR primers for classical and next-generation sequencing-based diversity studies. Nucleic Acids Res 41(1):e1 doi:10.1093/nar/gks808

Love MI, Huber W, Anders S (2014) Moderated estimation of fold change and dispersion for RNA-seq data with DESeq2. Genome Biol 15(12):550 doi:10.1186/s13059-014-0550-8

Lozupone C, Lladser ME, Knights D, Stombaugh J, Knight R (2011) UniFrac: an effective distance metric for microbial community comparison. ISME J 5(2):169-72 doi:10.1038/ismej.2010.133

Lu Y, Zhou G, Ewald J, Pang Z, Shiri T, Xia J, MicrobiomeAnalyst 2.0: comprehensive statistical, functional and integrative analysis of microbiome data, Nucleic Acids Research, Volume 51, Issue W1, 5 July 2023, Pages W310–W318, https://doi.org/10.1093/nar/gkad407

McMurdie PJ, Holmes S (2013) phyloseq: an R package for reproducible interactive analysis and graphics of microbiome census data. PLoS One 8(4):e61217 doi:10.1371/journal.pone.0061217

Murali A, Bhargava A, Wright ES (2018) IDTAXA: a novel approach for accurate taxonomic classification of microbiome sequences. Microbiome 6(1):140 doi:10.1186/s40168-018-0521-5

Oksanen J, Blanchet FG, Kindt R, et al. (2016) vegan: Community Ecology Package.

Paradis E, Schliep K (2019) ape 5.0: an environment for modern phylogenetics and evolutionary analyses in R. Bioinformatics 35(3):526-528 doi:10.1093/bioinformatics/bty633

Quast C, Pruesse E, Yilmaz P, et al. (2013) The SILVA ribosomal RNA gene database project: improved data processing and web-based tools. Nucleic Acids Res 41(Database issue):D590-6 doi:10.1093/nar/gks1219

Wickham H (2009) ggplot2: Elegant Graphics for Data Analysis. Springer-Verlag, New York

Yilmaz P, Parfrey LW, Yarza P, et al. (2014) The SILVA and "All-species Living Tree Project (LTP)" taxonomic frameworks. Nucleic Acids Res 42(Database issue):D643-8 doi:10.1093/nar/gkt1209

Supplementary Material

# 3 Supplementary Figures and Tables

## Supplementary Figures

**Fig S1 - Microbial variation during regeneration (continued). (A)** Relative abundances at the phylum level per individual head fragment during the different stages of regeneration, for two independent experiments. **(B)** Relative abundances at the phylum level per individual tail fragment during different stages of the regeneration, for two independent experiments. (RA: relative abundance, dpa; days post amputation, head 0 dpa *n* = 6, head 3 dpa *n* = 5, head 7 dpa *n* = 6, head 14 dpa *n* = 3, tail 0 dpa *n* = 4, tail 3 dpa *n* = 5, tail 7 dpa *n* = 6, tail 14 dpa *n* = 3) **(C)** Rarefaction curves for the samples in the regeneration experiment. **(D)** PCoA with 3th axis of variation of weighted UniFrac illustrating beta-diversity between the different regeneration stages in head and tail fragments, colored by regeneration stage. Image generated in MicrobiomeAnalyst (Dhariwal et al. 2017). **(E)** PCoA with 3th axis of variation of weighted UniFrac illustrating beta-diversity between the different regeneration stages in head and tail fragments, colored by fragment. Image generated in MicrobiomeAnalyst (Dhariwal et al. 2017). **(F)** Relative abundance of genera found in planarian medium. The barplot represents 1 sample.

**Fig S2 - Microbial variation in *S. mediterranea* due to an alternative diet (continued). (A)** Average total body surface (mm²) per week in starved, liver-fed and egg-fed worms. **(B)** Percentage of worms that underwent asexual fission in liver, egg-fed and liver-fed worms over the course of 8 weeks (starved *n* = 6, liver-fed *n* = 6, egg-fed *n* = 6). **(C)** Number of sequencing reads from pieces of liver and egg, compared to the average number of reads in worm and medium samples. **(D)** Genera assigned to the reads of the pieces of liver and egg. **(E)** Average relative abundances at order level at the different time-points for the different conditions, t_0_ = starting point, t_1_ = 1 week, t_2_ = 2 weeks, t_3_ = 3 weeks, t_4_ = 4 weeks, t_8_ = 8 weeks. The orders are grouped to phyla by colour and the size of the bubble corresponds to the relative abundance. (W: worms, M: exposure medium that came into contact with worms, RA: relative abundance, t_0_ M *n* = 4, t_0_ W *n* = 4, t_1_ liver-fed *n* = 3, t_1_ egg-fed *n* = 3, t_3_ liver-fed *n* = 3, t_3_ egg-fed *n* = 3, t_4_ liver-fed *n* = 3, t_4_ egg-fed *n* = 3, t_8_ liver-fed *n* = 3, t_8_ egg-fed *n* = 3) **(F)** Rarefaction curves for the samples exposed to an alternative diet.

**Fig S3 - Differentially abundant genera and rarefaction curves after external exposures for *S. mediterranea* (A)** Plot showing the differentially abundant genera in *S. mediterranea* exposed to gentamycin and vancomycin (p-value < 0.05). **(B)** Plot showing the differentially abundant genera in *S. mediterranea* exposed to cadmium and MMS (p-value < 0.05). **(C)** Rarefaction curves for gentamycin and vancomycin exposed worms, and their corresponding controls. **(D)** Rarefaction curves for cadmium and MMS exposed worms, and their corresponding controls.

**Fig S4 – Rarefaction curves for adult non-exposed worms at different timepoints (A)** For the samples of 2017. **(B)** For the samples of 2018 (1). **(C)** For the samples of 2018 (2). **(D)** For the samples of 2019.

**Fig S5 - Differentially abundant ASVs and common microbiome for *S. mediterranea* originating from 3 different laboratories (A)** Volcanoplots showing the differentially abundant ASVs with their corresponding genera in *S. mediterranea* originating from Lab 1, Lab 2 and Lab 3 (red: p-value < 0.001 and -1 > log_2_FC > 1). **(B)** Venn diagram showing the number of common ASVs and corresponding genera in *S. mediterranea* originating from Lab 1, Lab 2 and Lab 3. (Unass.: unassigned, FC: fold change) **(C)** Rarefaction curves for the samples originating from 3 different laboratories.

**Fig S6 - Differentially abundant ASVs and common microbiome for *S. mediterranea, G. tigrina* and *Dugesia* sp.. (A)** Volcanoplots showing the differentially abundant ASVs with their corresponding genera in *S. mediterranea*, *G. tigrina* and *Dugesia* sp. (red: p-value < 0.001 and -1 > log_2_FC > 1). **(B)** Venn diagram showing the number of common ASVs and corresponding genera in *S.* *mediterranea*, *G. tigrina* and *Dugesia* sp.. (Unass.: unassigned, FC: fold change) **(C)** Rarefaction curves for the samples for the different planarian species.

**Fig S7 - Exploratory analyses after functional prediction of the microbiome of intact (adult) and regenerating (0 dpa, 3 dpa, 7 dpa and 14 dpa; head and tails) worms. (A)** Principal component analysis based on the predicted features. The samples are colored by timing of the experiment (0 dpa, 3 dpa, 7 dpa, 14 dpa and intact). **(B)** Barplot showing the functional diversity of each sample, grouped by KEGG metabolism categories. **(C)** Top 25 discriminatory features for adult vs regenerating worm based on a Linear Discriminant Analysis Effect Size (LEfSe) based on the functional predictions of the microbiome of intact (adult) and regenerating (0 dpa, 3 dpa, 7 dpa and 14 dpa; head and tails). **(D)** Top 25 discriminatory features obtained after LEfSe based on the factor ‘timing’ (0 dpa, 3 dpa, 7 dpa, 14 dpa and none/intact). The heatmap to the right shows the abundance of the feature in the corresponding ‘timing’ category. (p-value cutoff 0.1, FDR-adjusted; log LDA score > 2.0). Images generated in MicrobiomeAnalyst (Dhariwal et al. 2017), using the KEGG database (Kanehisa et al. 2000, Kanehisa 2019, Kanehisa et al. 2023).

**Fig S8 - Exploratory analyses after functional prediction of the microbiome of control, Cd and MMS exposed worms. (A)** Barplot showing the functional diversity of each sample, grouped by KEGG metabolism categories. **(B)** Linear Discriminant Analysis Effect Size (LEfSe) based on the functional (p-value cutoff 1, FDR-adjusted; log LDA score > 2.0). Images generated in MicrobiomeAnalyst (Dhariwal et al. 2017), using the KEGG database (Kanehisa et al. 2000, Kanehisa 2019, Kanehisa et al. 2023).

**Fig S9 – Localization of the planarian microbiome. (A)** Scanning electron micrograph of a worm, zoomed in at the gut region. The blue line indicates an approximation of the outline of the planarian gut. **(B)** Fluorescent in situ hybridisation micrograph generated with probes complementary for all bacteria. Left: schematic overview of the worm. Middle: FISH generated with probes complementary to all bacteria (Ph: pharnynx, A: anterior part of the worm, P: posterior part of the worm). Right: negative control (NC). Scale bar represents 100 µm. The images are a projection of the signal of all layers. The grey line indicates an approximation of the outline of the planarian gut.

## Supplemental tables

**Table S1 – Bacteria cultivated from *S. mediterranea*, identified via Sanger sequencing.** Based on color, shape and appearance, 32 isolates were selected and identified.

| **ID** | **Top-hit taxon** | **Top-hit strain** | **Similarity (%)** | **Top-hit taxonomy** |
| --- | --- | --- | --- | --- |
| A09 | *Chryseobacterium phosphatilyticum* | ISE14 | 96.85 | Bacteria;Bacteroidetes;Flavobacteriia;Flavobacteriales;Weeksellaceae;*Chryseobacterium* |
| A10 | *Chryseobacterium phosphatilyticum* | ISE14 | 97.22 | Bacteria;Bacteroidetes;Flavobacteriia;Flavobacteriales;Weeksellaceae;*Chryseobacterium* |
| A11 | *Microbacterium oxydans* | DSM 20578 | 97.92 | Bacteria;Actinobacteria;Actinomycetia;Microbacteriales;Microbacteriaceae;*Microbacterium* |
| A12 | *Acidovorax soli* | DSM 25157 | 98.79 | Bacteria;Proteobacteria;Betaproteobacteria;Burkholderiales;Comamonadaceae;*Acidovorax* |
| B09 | *Paucibacter oligotrophus* | CHU3 | 96.29 | Bacteria;Proteobacteria;Betaproteobacteria;Burkholderiales;Sphaerotilaceae;*Paucibacter* |
| B10 | *Chryseobacterium phosphatilyticum* | ISE14 | 97.38 | Bacteria;Bacteroidetes;Flavobacteriia;Flavobacteriales;Weeksellaceae;*Chryseobacterium* |
| B11 | *Bosea robiniae* | DSM 26672 | 96.72 | Bacteria;Proteobacteria;Alphaproteobacteria;Rhizobiales;Boseaceae;*Bosea* |
| B12 | *Micrococcus endophyticus* | YIM 56238 | 96.72 | Bacteria;Actinobacteria;Actinomycetia;Micrococcales;Micrococcaceae;*Micrococcus* |
| C09 | *Acidovorax soli* | DSM 25157 | 94.20 | Bacteria;Proteobacteria;Betaproteobacteria;Burkholderiales;Comamonadaceae;*Acidovorax* |
| C10 | *Paucibacter oligotrophus* | CHU3 | 94.72 | Bacteria;Proteobacteria;Betaproteobacteria;Burkholderiales;Sphaerotilaceae;*Paucibacter* |
| C11 | *Chryseobacterium phosphatilyticum* | ISE14 | 97.51 | Bacteria;Bacteroidetes;Flavobacteriia;Flavobacteriales;Weeksellaceae;*Chryseobacterium* |
| C12 | *Pseudomonas vanderleydeniana* | RW8P3 | 67.03 | Bacteria;Proteobacteria;Gammaproteobacteria;Pseudomonadales;Pseudomonadaceae;*Pseudomonas* |
| D09 | *CP022960_s* | NS1 | 96.06 | Bacteria;Proteobacteria;Gammaproteobacteria;Pseudomonadales;Pseudomonadaceae;*Pseudomonas* |
| D10 | *CP022960_s* | NS1 | 97.17 | Bacteria;Proteobacteria;Gammaproteobacteria;Pseudomonadales;Pseudomonadaceae;*Pseudomonas* |
| D11 | *JTGD_s* | F7 | 96.46 | Bacteria;Proteobacteria;Alphaproteobacteria;Rhizobiales;Phyllobacteriaceae;*Mesorhizobium* |
| D12 | *Variovorax beijingensis* | 502 | 98.75 | Bacteria;Proteobacteria;Betaproteobacteria;Burkholderiales;Comamonadaceae;*Variovorax* |
| E09 | *Flectobacillus rhizosphaerae* | JC289 | 93.06 | Bacteria;Bacteroidetes;Cytophagia;Cytophagales;Spirosomaceae;*Flectobacillus* |
| E10 | *Paucibacter oligotrophus* | CHU3 | 94.97 | Bacteria;Proteobacteria;Betaproteobacteria;Burkholderiales;Sphaerotilaceae;*Paucibacter* |
| E11 | *Pedobacter ghigonis* | Marseille-Q2390 | 97.47 | Bacteria;Bacteroidetes;Sphingobacteriia;Sphingobacteriales;Sphingobacteriaceae;*Pedobacter* |
| E12 | *Variovorax paradoxus* | NBRC 15149 | 96.43 | Bacteria;Proteobacteria;Betaproteobacteria;Burkholderiales;Comamonadaceae;*Variovorax* |
| F09 | *Paucibacter oligotrophus* | CHU3 | 95.63 | Bacteria;Proteobacteria;Betaproteobacteria;Burkholderiales;Sphaerotilaceae;*Paucibacter* |
| F10 | *Microbacterium algeriense* | G1 | 96.48 | Bacteria;Actinobacteria;Actinomycetia;Microbacteriales;Microbacteriaceae;*Microbacterium* |
| F11 | *Microbacterium algeriense* | G1 | 97.47 | Bacteria;Actinobacteria;Actinomycetia;Microbacteriales;Microbacteriaceae;*Microbacterium* |
| F12 | *Microbacterium oxydans* | DSM 20578 | 95.01 | Bacteria;Actinobacteria;Actinomycetia;Microbacteriales;Microbacteriaceae;*Microbacterium* |
| G09 | *Acidovorax soli* | DSM 25157 | 97.36 | Bacteria;Proteobacteria;Betaproteobacteria;Burkholderiales;Comamonadaceae;*Acidovorax* |
| G10 | *Bosea spartocytisi* | SSUT16 | 95.93 | Bacteria;Proteobacteria;Alphaproteobacteria;Rhizobiales;Boseaceae;*Bosea* |
| G11 | *Sphingopyxis chilensis* | S37 | 97.01 | Bacteria;Proteobacteria;Alphaproteobacteria;Sphingomonadales;Sphingomonadaceae;*Sphingopyxis* |
| G12 | *Pseudomonas putida* | NBRC 14164 | 98.32 | Bacteria;Proteobacteria;Gammaproteobacteria;Pseudomonadales;Pseudomonadaceae;*Pseudomonas* |
